# Tracing the Source of Campylobacteriosis

**DOI:** 10.1371/journal.pgen.1000203

**Published:** 2008-09-26

**Authors:** Daniel J. Wilson, Edith Gabriel, Andrew J. H. Leatherbarrow, John Cheesbrough, Steven Gee, Eric Bolton, Andrew Fox, Paul Fearnhead, C. Anthony Hart, Peter J. Diggle

**Affiliations:** 1 Department of Maths and Statistics, Lancaster University, Lancaster, United Kingdom; 2 Department of Medicine, Lancaster University, Lancaster, United Kingdom; 3 Faculty of Veterinary Science, University of Liverpool, Leahurst, Neston, United Kingdom; 4 Preston Microbiology Services, Royal Preston Hospital, Lancashire Teaching Hospitals NHS Foundation Trust, Preston, United Kingdom; 5 Manchester Medical Microbiology Partnership, Manchester Royal Infirmary, Manchester, United Kingdom; 6 Division of Medical Microbiology, School of Infection and Host Defence, University of Liverpool, Liverpool, United Kingdom; University of Toronto, Canada

## Abstract

*Campylobacter jejuni* is the leading cause of bacterial gastro-enteritis in the developed world. It is thought to infect 2–3 million people a year in the US alone, at a cost to the economy in excess of US $4 billion. *C. jejuni* is a widespread zoonotic pathogen that is carried by animals farmed for meat and poultry. A connection with contaminated food is recognized, but *C. jejuni* is also commonly found in wild animals and water sources. Phylogenetic studies have suggested that genotypes pathogenic to humans bear greatest resemblance to non-livestock isolates. Moreover, seasonal variation in campylobacteriosis bears the hallmarks of water-borne disease, and certain outbreaks have been attributed to contamination of drinking water. As a result, the relative importance of these reservoirs to human disease is controversial. We use multilocus sequence typing to genotype 1,231 cases of *C. jejuni* isolated from patients in Lancashire, England. By modeling the DNA sequence evolution and zoonotic transmission of *C. jejuni* between host species and the environment, we assign human cases probabilistically to source populations. Our novel population genetics approach reveals that the vast majority (97%) of sporadic disease can be attributed to animals farmed for meat and poultry. Chicken and cattle are the principal sources of *C. jejuni* pathogenic to humans, whereas wild animal and environmental sources are responsible for just 3% of disease. Our results imply that the primary transmission route is through the food chain, and suggest that incidence could be dramatically reduced by enhanced on-farm biosecurity or preventing food-borne transmission.

## Author Summary

*C. jejuni* is a bacterium commonly found in the guts of birds and mammals. In humans, it is responsible for causing more gastro-enteritis than any other identified bacterial species. Humans may contract campylobacter from a variety of sources. Eating raw or undercooked meat or poultry, and poor food hygiene that leads to cross-contamination of uncooked food, can cause human disease. However, humans may be exposed to the feces of infected wild animals, and campylobacter can survive in water. Contamination of drinking water can lead to outbreaks, and previous genetic studies have suggested that livestock are not the principal source of human infection. We extracted campylobacter DNA from patients and compared it to campylobacter DNA found in livestock, wild animals, and the environment. We developed a new evolutionary model to identify the most probable source populations. In 97% of cases, we identified chicken, cattle, or sheep as the source of infection. Very few cases were attributable to campylobacter found in wild animals or the environment. Our results imply that the primary transmission route is the food chain and also add new impetus to measures that reduce infection in livestock and prevent food-borne transmission.

## Introduction

*Campylobacter* is the most commonly identified cause of bacterial gastro-enteritis in the developed world [1],[2],[3]. Infection can lead to serious sequelae such as Guillain-Barré syndrome and reactive arthritis [4]. Of the species pathogenic to humans, 90% of disease is caused by *C. jejuni* and most of the rest by *C. coli* [5]. Both species are zoonotic pathogens with wide host ranges including farm animals (cattle, sheep, poultry, pigs) and wild animals (birds and mammals) [1],[6],[7]. The bacterium thrives at 37–42C in the mammalian and avian gut, but survives longest *ex vivo* in cold, dark, moist environments. *Campylobacter* is routinely isolated from fresh and marine water sources, and sewage [8].

Epidemiological studies have demonstrated a link with exposure to contaminated food. Handling and eating raw and undercooked poultry have consistently been shown to be important risk factors. Case-control studies show that red meat and seafood are risk factors, as are eating at restaurants and barbecues, and drinking raw milk [9],[10]. However, food is not the only danger, and some studies have shown that regularly eating poultry and red meat in the home actually has a protective effect [10]. Water, particularly when untreated, can present a threat. Incidence of campylobacteriosis is typically sporadic, but outbreaks do occur that can often be traced to contamination of the water supply [11]–[13]. Some authors have suggested that the strong seasonal variation in sporadic disease, which rises sharply in spring and peaks in summer, bears the hallmark of water-borne diseases such as cryptosporidiosis [8],[9],[14].

DNA-based methods of typing *C. jejuni* have the potential to resolve the controversy surrounding the origin of infection, but have thus far failed to do so. *C. jejuni* isolated from humans show considerable genetic overlap with meat and poultry isolates [15],[16],[17]. However, a model-based approach that includes disparate sources is needed. Although *C. jejuni* genotypes do show some host association, the population is not strongly structured into differentiated clusters, so predicting host from genotype is challenging [6]. Phylogenetic approaches to tracing the source of infection have suggested that human isolates are more closely related to *C. jejuni* found in non-livestock than livestock [18]. But recombination is frequent in *C. jejuni* [19], which means that a single phylogenetic tree is not an appropriate way to represent the ancestral history of a collection of *C. jejuni* genomes [20].

Here we report a systematic study of 1,231 cases of *C. jejuni* infection in Lancashire, England, which we have DNA-sequenced using multi-locus sequence typing [21] (MLST). We infer the source of infection of each patient by comparison to 1,145 animal and environmental *C. jejuni* isolates collated from previous studies in livestock, wild animals and the environment [7], [16], [21]–[28], using a novel population genetics approach that models DNA sequence evolution and zoonotic transmission. We treat the animal and environmental reservoirs of *C. jejuni* as populations between which there may be gene flow (migration). Within these populations the bacteria evolve through *de novo* mutation and horizontal gene transfer (recombination). We estimate the amount of mutation, migration and recombination, and use these estimates to assign probabilistically each human case to one of the source populations. From these population assignments we estimate the total amount of human disease attributable to each source.

## Results

### Diversity and Differentiation in *C. jejuni* Populations

We observed 256 distinct genotypes (or sequence types, STs) in the 1,231 human isolates. The frequency of genotypes was highly skewed, with 20% of STs accounting for 80% of cases. The two most frequent genotypes (STs 21 and 257) made up a quarter of cases alone, while 182 genotypes were observed once only. There were 375 distinct STs in the 1,145 animal and environmental isolates, and overlap with the human genotypes was extensive. Six STs featured in both the human and non-human lists of ten most common genotypes (STs 19, 21, 45, 50, 53, 61). However, nearly a quarter of human cases (278) exhibited genotypes exclusive to humans (189 STs), most of those at low frequency. The most abundant human-specific genotypes were ST 572 (14 cases) and ST 584 (19 cases). Over a third of non-human isolates (440) possessed genotypes absent from our human sample (308 STs).

Certain genotypes common in non-human isolates were host-restricted to varying degrees. For example, ST 403 was the most prevalent in pigs (5/30 isolates), but absent from other non-human groups. ST 61 is common in ruminants (cattle and sheep) but rare in other groups, while ST 45 was frequent in all the non-human reservoirs except pig and sand. At the level of individual loci, many alleles were frequently observed in a range of animal and environmental sources. Because of the large overlap in genetic variation between *C. jejuni* reservoirs, our method utilizes differences in gene frequency, rather than allele presence or absence *per se*. By pooling samples of *C. jejuni* from similar sources (*e.g.* chicks and chicken meat/offal) and several studies, we intended to improve inference by increasing sample size. See Table S1 for details. However, by combining samples this way, we implicitly assumed that within each group (chicken, cattle, sheep, pig, bird, rabbit, sand and water) gene frequencies are consistent across sources and across studies. To test this assumption, and to quantify genetic differentiation between groups, we used analyses of molecular variance (AMOVA [29]).

AMOVA quantifies genetic differentiation within and between groups using Φ-statistics, which measure the correlation in gene frequencies within sub-populations relative to the total population. A smaller value of Φ indicates lower genetic differentiation between the populations. Table 1 shows Φ*_SG_*, the genetic differentiation within each group (*e.g.* chicken) between isolates of a different sub-group: *i.e.* source type (*e.g.* chick *vs* chicken meat/offal) or published study. Except for the sand group, there was significant heterogeneity within the groups that comprised more than one source type or study. Genetic differentiation between sub-groups ranged from 2.4% (cattle) to 23.2% (pig). This suggests that gene frequencies vary significantly between similar sources and between different studies of the same source.

**Table 1:** Genetic differentiation within and between groups.

Genetic differentiation (Φ*_SG_*) within groups								
	CHICKEN	CATTLE	SHEEP	PIG	BIRD	RABBIT	SAND	WATER
Φ*_SG_*	**11.8%**	**2.4%**	**11.2%**	**23.2%**	**9.8%**	-	0.0%	-
*p*	0.001	0.003	0.001	0.003	0.003	-	0.969	-

Genetic differentiation (Φ*_GT_*) between groups, over and above within-group differentiation								
Φ*_GT_*\*p*	CHICKEN	CATTLE	SHEEP	PIG	BIRD	RABBIT	SAND	WATER
CHICKEN	-	0.001	0.003	0.001	0.319	0.807	0.150	0.270
CATTLE	**4.4%**	-	0.182	0.001	0.049	0.170	0.053	0.001
SHEEP	**4.4%**	0.1%	-	0.006	0.145	0.414	0.102	0.544
PIG	**18.6%**	**24.8%**	**26.2%**	-	0.108	0.164	0.089	0.001
BIRD	0.0%	**9.6%**	8.1%	13.7%	-	0.756	0.001	0.293
RABBIT	0.0%	3.1%	0.9%	11.8%	0.0%	-	0.001	0.353
SAND	4.0%	8.3%	7.6%	22.2%	**4.7%**	**7.4%**	-	0.001
WATER	0.0%	**12.7%**	11.6%	**13.2%**	0.0%	0.3%	**11.3%**	-

Total genetic differentiation between isolates from two different groups, Φ*_ST_*, equals approximately 1−(1−Φ*_SG_*)(1−Φ*_GT_*) where Φ*_SG_* represents an average for the two groups. Significant Φ-statistics are printed in bold.

In order to assign human cases to source populations with any degree of accuracy, there must be genetic differentiation between the groups, *over and above* within-group heterogeneity. We estimated that quantity, Φ*_GT_*, using nested AMOVA between pairs of groups. Table 1 shows the results. For each pair of groups, Φ*_GT_* is displayed below the diagonal and the associated *p*-value above the diagonal. All groups were significantly differentiated from at least one other group in this way, with Φ*_GT_* ranging from 4.4% (chicken *vs* cattle) to 26.2% (sheep *vs* pig). However, there were some major groups that were not significantly differentiated. Notably, Φ*_GT_* was 0.1% for cattle *vs* sheep (*p* = 0.182), which suggested it would be difficult to distinguish human cases attributable to these two groups.

The preliminary analyses of the animal and environmental *C. jejuni* isolates presented several potential concerns. There was significant variation in gene frequencies within groups, probably caused by the heterogeneous nature of the studies from which the non-human isolates were drawn, and the inherently stochastic nature of the epidemic process. This could distort the gene frequency information upon which source assignment relies, and cause higher than expected linkage disequilibrium between loci. AMOVA also showed that genetic differentiation between groups was weak in some cases. Within-group heterogeneity could therefore obscure or potentially distort the signal of differentiation between groups. Another concern was that the large differences in sample size between non-human groups, which reflect a tendency among researchers to preferentially sample certain hosts, could bias the source assignment. To investigate the sensitivity of our method to these effects, and to test its robustness to violating the assumption of homogeneous mixing within groups, we performed empirical cross-validation.

### Empirical Cross-Validation

Traditional methods that can assign large numbers of individuals to populations based on their genotype tend to assume that loci provide independent sources of information [30],[31]. In other words, they assume that gene frequencies between loci are uncorrelated in the source populations. While this simplifying assumption is computationally convenient, it may not be appropriate for *C. jejuni* because of appreciable linkage disequilibrium between MLST loci [19]. Therefore we developed two models, one in which loci were assumed to be *unlinked* (*i.e.* independent, or in linkage equilibrium) and another in which loci were *linked* (*i.e.* in linkage disequilibrium); see Materials and Methods. We used empirical cross-validation to scrutinize both.

In each of 100 simulations, we removed the source information from half the non-human isolates, chosen at random. These we termed the pseudo-human cases. We used our *unlinked* and *linked* models to assign the source of the pseudo-human cases using the other non-human isolates. Table 2 shows that the two models differed considerably in performance. On average, the *unlinked* model correctly assigned 52% of the pseudo-human cases (using the rule that each case is assigned to its most probable source), whereas the *linked* model correctly assigned 64%. The *linked* model was well-calibrated in the sense that its estimated success rate was 64% on average, whereas the *unlinked* model grossly over-estimated its success rate (82% on average). We used a number of performance indicators to measure the ability of each model to correctly estimate the total proportion of pseudo-human cases attributable to a given source (see Table 2). The parameter estimates obtained by using the *linked* model generally exhibited lower bias and smaller variance (measured by root mean squared error, RMSE) than those obtained using the *unlinked* model. The *linked* model also out-performed the *unlinked* model in coverage, which we defined as the number of simulations, out of 100, in which the 95% credible interval for the proportion of cases attributable to a given source enveloped the true value. For seven out of eight groups, the *linked* model obtained the target coverage of 95 or above. Coverage was 93 for chicken; the small negative bias suggests this may have been caused by slightly under-estimating the proportion of pseudo-human cases attributable to chicken.

**Table 2:** Performance of the models during empirical cross-validation.

		Unlinked model	Linked model
**Proportion of isolates correctly assigned**	Actual	0.52	0.64
	Predicted	0.82	0.64
**Bias**	Chicken	−0.10	−0.03
	Cattle	−0.13	0.00
	Sheep	0.20	0.03
	Pig	0.01	0.00
	Bird	0.00	−0.01
	Rabbit	0.00	0.01
	Sand	−0.01	0.00
	Water	0.03	0.00
**RMSE**	Chicken	0.11	0.04
	Cattle	0.14	0.07
	Sheep	0.21	0.07
	Pig	0.01	0.01
	Bird	0.03	0.02
	Rabbit	0.02	0.02
	Sand	0.01	0.01
	Water	0.05	0.01
**Coverage**	Chicken	12	93
	Cattle	19	97
	Sheep	5	97
	Pig	76	99
	Bird	86	99
	Rabbit	73	100
	Sand	93	99
	Water	84	99

The unlinked and linked models are defined in the Methods. The predicted proportion of isolates correctly assigned assumes that isolates are assigned to their most probable source *a posteriori*. Bias, RMSE (root mean squared error) and coverage are reported for the proportion of isolates estimated to originate from each source. Coverage was defined as the number of simulations, out of 100, in which the true proportion fell inside the 95% credible interval.

In the empirical cross-validation the *linked* model performs well despite the potential concerns due to heterogeneity within the animal and environmental groups, and differences in sample size. Most importantly, it is well-calibrated in assigning isolates to source populations, and estimating the overall proportion of cases attributable to each source. In contrast, the *unlinked* model assigns fewer isolates to source populations correctly, and is very poorly calibrated. This underlines the importance of adequately modeling recombination in the study of pathogen evolution. Clearly the computational efficiency gains made by assuming independent inheritance among loci in the *unlinked* model are out-weighed by its poor performance. Therefore we use the *linked* model for our analysis proper.

### Tracing the Source of Human Cases

**Figure 1:**
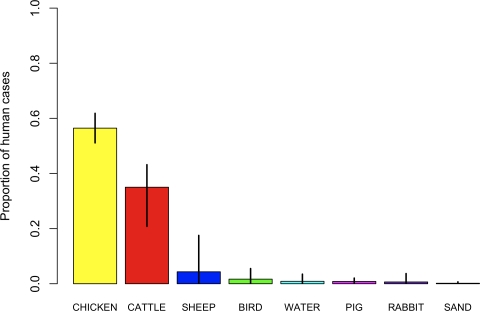
Estimated proportion of human cases attributable to animal and environmental sources. Error bars indicate the 95% credible interval for each source.

We applied our novel method to the 1,231 newly-sequenced human isolates from Lancashire, England. For every case, the assignment probability was calculated for each source population (chicken, cattle, sheep, pig, bird, rabbit, sand, water), and the total proportion of cases attributable to each source was estimated. We found that the vast majority (96.6%) of human cases are attributable to populations of *C. jejuni* carried by livestock (95% credible interval 92.7–98.8%) as opposed to wild animals (2.3%) or environmental isolates (1.1%). Figure 1 shows a breakdown of attribution by source; errors bars indicate the 95% credible intervals. We estimated that chicken is the source of infection in the majority (56.5%) of cases (95% C.I. 51.1–61.8%, see Table S2), followed by cattle (35.0%) and sheep (4.3%). The 95% credible intervals were wider for cattle (20.8–43.2%) and sheep (0.1–17.5%) than other groups, which reflects the greater difficulty in distinguishing these populations of *C. jejuni* from one another. We found that pig is unlikely to be the source of *C. jejuni* infection in humans (0.8% of cases).

Of the two groups of wild animals we studied, bird and rabbits, there was somewhat more support for a wild bird origin of human *C. jejuni* (1.7%) than rabbit (0.6%), although the credible intervals (0.1–5.5% and 0.0–3.7% respectively) were largely overlapping. There was very little support for an environmental origin of human infections. Even so, the results suggested that infection with *C. jejuni* found in environmental water sources was more likely (0.9%) than infection with *C. jejuni* isolated from bathing beaches (0.2%), which was the least likely of all sources. Overall, the analysis reported that with 98.3% probability, chicken is the primary, and cattle the secondary source of human infections in our study.

**Figure 2:**
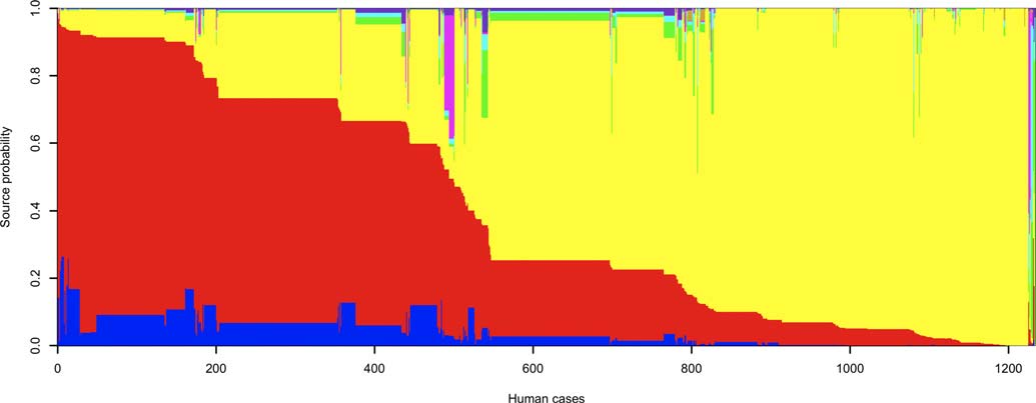
Probability of source for human cases. The source probability for 1,231 human cases (vertical columns) is depicted for Chicken (yellow), Cattle (red), Sheep (blue), Pig (pink), Bird (green), Rabbit (purple), Sand (beige) and Water (cyan). The isolates have been ordered horizontally to aid visualization.

The posterior probability of source of infection was estimated for each patient in our study; Figure 2 illustrates the results. The source populations are color-coded as in Figure 1. Cases are arranged horizontally, and the vertical column space occupied by each color represents the posterior probability of infection from that source. The dominant color in any column indicates the most likely source for a particular case. The principal distinction in human cases is between those attributed to chicken versus ruminants (cattle and sheep). Most cases lie on a continuum between assignment to ruminants and to chicken. The existence of this continuum, as opposed to a clear separation, emphasizes the overlap in genotypes between these source populations, and the advantage of a probabilistic approach to assignment. Some common genotypes were strongly assigned to ruminants (*e.g.* ST 48, 86 cases, posterior probability [*Pr*] = 0.91) and others to chicken (ST 104, 64 cases, *Pr* = 0.93). But within ruminants, it is harder to distinguish cattle from sheep sources. This is borne out by the strong correlation among cases between cattle and sheep assignment probabilities (*ρ* = 0.80).

In some cases, there is moderate or strong support for a source that is generally found to be rare. For example, there were six cases of ST 403, with a moderately high assignment probability to pig of 0.37. Except for the human isolates, we observed ST 403 only in the pig population. However, because the evidence overall suggests that pig is an unlikely source of infection for humans, and because of the genetic similarity to cattle genotypes (*e.g.* ST 933), it is marginally more likely under the model that cattle is the source of these cases (*Pr* = 0.46). Although it is most probable, on a case-by-case basis, that the source of infection was cattle, when considered together we would expect the source of infection to have been cattle in 2.7 of those cases, pig in 2.2 cases and chicken in 0.6 cases. Another example of this phenomenon is found in birds. There are 28 cases, of which ST 508 was the most common genotype, with an assignment probability to birds greater than 10%, but a larger assignment probability to another source, usually chicken. On an individual basis none of these cases would be assigned to birds, but taken together we estimate that the source of infection was birds in 5.6 of them, chicken in 10.6, cattle in 5.5 and water in 3.8. Overall, the source probabilities in Figure 1 and Table S2 suggest that of the 1,231 human cases, the source of infection was chicken in 696.6 cases, cattle in 432.1, sheep in 53.5, bird in 20.5, water in 10.9, pig in 10.3, rabbit in 7.9 and sand in 2.2.

Sometimes it is useful to assign a case to a single source, in which case the optimal strategy is to attribute it to the source with highest assignment probability *a posteriori*. We estimate that 76.5% of human cases would be correctly assigned by this procedure. Earlier we showed that this quantity, which is the average maximum source attribution probability per case, was well-calibrated during empirical cross-validation. When cases are assigned to sources in this fashion, most are assigned to chicken (722) or cattle (503). None are assigned to sheep, because ruminant-associated isolates are assigned preferentially to cattle. A small number are assigned to pig, bird and water (three in each case). For example, STs 1286, 1927 and 2973 were the genotypes most strongly assigned to environmental water, pig and wild bird respectively (*Pr* = 0.58, 0.65, 0.87). Interestingly, all three genotypes were human-specific, and each was found in a single patient only. In the case of ST 1286, there was also considerable support for a wild bird origin (*Pr* = 0.35), an observation that may reflect the low genetic differentiation detected between these sources (Table 1). Table S3 gives a detailed breakdown of source attribution probabilities by sequence type.

### Robustness to Within-Group Heterogeneity

Our collection of animal and environmental isolates which we collated from previously-published studies [7], [16], [21]–[28] were non-ideal in several respects. AMOVA revealed significant variation between isolates from the same group that originated in different sub-groups – *i.e.* different source types or studies. Such genetic structuring will cause higher than expected linkage disequilibrium within groups, and may distort the gene frequencies upon which source attribution relies. Although empirical cross-validation showed that the *linked* model was robust to these effects, the full extent of the difficulty caused by within-group heterogeneity may have been masked because individual isolates were assigned to the pseudo-human class independently, and without reference to their sub-group. Therefore we performed additional simulations in which whole sub-groups of isolates were removed, and the human isolates re-analyzed based on the reduced set of animal and environmental isolates. In each simulation, we removed at least 20% of the animal and environmental isolates, 24.5% on average. Figure S2 illustrates the simulation scheme and contrasts it to the simulations used in empirical cross-validation.

Our main conclusions are robust to genetic heterogeneity within the source populations. Figure S3 summarizes the analysis of robustness by plotting the point estimate and 95% credible interval of various parameters based on the 100 simulations and the full data. In all of the 100 simulated datasets analyzed, chicken was found to be the primary source of human infections. Figure S3A shows that in the majority of simulations, chicken accounted for more than 50% of human disease. The conclusion that ruminants are the second most important source of human infection was also supported by the analysis (Figure S3B). Despite the low genetic differentiation between cattle and sheep, as witnessed by the AMOVA results, the finding that cattle account for considerably more disease than sheep is surprisingly robust to re-sampling the non-human isolates. In Figure S3C, the posterior median (rather than the mean) is used to illustrate that in 87 simulations, a greater proportion of human cases were attributed to cattle than to sheep.

The greatest effect of the re-sampling of non-human isolates was seen in the proportion of human cases attributed to the bird group. Figure S3D shows that in a minority of simulations (16 out of 100), the proportion of cases attributed to birds leapt ten-fold to around 20%, promoting it to the second or third most important source, compared to fourth in the analysis of the full data. Of these 16 simulations, there was a significantly lower number of bird isolates (*p* = 0.005) and a significantly higher number of chicken isolates (*p* = 0.040) compared to the other simulations, the relevance of which is that the chicken and bird groups were shown by AMOVA to exhibit extremely low genetic differentiation (Table 1). While these results demonstrate that more intense sampling of the smaller groups, particularly birds, is highly desirable, our main conclusions are supported by the vast majority of re-sampled datasets, indicating a satisfactory level of robustness to within-group heterogeneity.

### Existence of Undiscovered Source Populations

A tacit assumption in our study, and in the ongoing sampling of *C. jejuni* populations, is that the major reservoirs have been identified. However, if a major source of human disease were undiscovered, we would expect to see an excess of genotypes unique to humans. In our study we observed 189 genotypes unique to humans. Of the 1,231 human cases, 278 possessed genotypes absent in the non-human isolates, but most of these (238 cases) were re-assortments of alleles or allele fragments that were present in the non-human isolates. In 254 cases, they differed at three loci or fewer to a non-human isolate. Out of 531 single nucleotide polymorphisms in humans, 40 were absent from the non-human samples. Of those, all were rare except an adenosine at nucleotide 448 in the *glnA* locus (12 copies), and a cytosine at nucleotide 93 in the *tkt* locus (13 copies). Two human-specific STs (572 and 584) had appreciable frequency (14 and 19 cases respectively).

It is difficult to quantify exactly what would constitute an excess of genotypes unique to humans. We employed a re-sampling procedure to compare the number of unique genotypes in human isolates compared to other groups, controlling for sample size. When sets of human isolates were drawn, equal in size to the number of chicken isolates (515), we observed fewer unique genotypes on average among human isolates (104.4) than among chicken isolates (153), where uniqueness was determined by reference to the “pool” of other non-human and non-chicken isolates. The same pattern was observed when comparing humans to cattle and birds, but not sheep (Figure S4A). Sheep isolates are genetically similar to cattle, which may explain why humans exhibit no more unique genotypes than do sheep. The observation that cattle isolates appear to exhibit relatively more unique genotypes than sheep suggests there might be an effect of sample size, or that sheep isolates are a subset of cattle isolates.

A second re-sampling procedure was designed to emulate the status of humans as a sample of isolates drawn from the putative source populations. Taking each non-human group in turn, half the isolates were removed, leaving the other half in the pool, and the number of genotypes unique to the removed isolates was calculated. A set of human isolates was drawn of equal number, and the number of unique genotypes calculated relative to the same pool. The whole procedure was repeated 100 times. If major source populations remained to be discovered, or if humans acted as a reservoir of *C. jejuni* rather than a terminus in the transmission chain, then an excess of genotypes unique to humans would be expected. However, in these simulations the distribution of the number of genotypes unique to humans and the non-human groups overlapped to a great extent (Figure S4B). Therefore while the more abundant STs and SNPs unique to humans deserve further attention, on the whole there is little indication that another major, genetically distinct, reservoir of human infection remains undiscovered.

## Discussion

Our results show that livestock are the principal source of *C. jejuni* infection in Lancashire, England. The vast majority of those human infections can be attributed to populations of *C. jejuni* found in chicken and cattle. These findings immediately lend weight to the suggestion that the incidence of campylobacteriosis in humans could be significantly reduced by intervention strategies targeted at livestock [32],[33], chiefly the strict enforcement of on-farm biosecurity measures including disinfecting farm premises and water supplies, restricting access to livestock to essential personnel, minimizing the use of invasive practices such as thinning in chickens, securing premises from wild birds and mammals, and protecting food supplies from bacterial contamination.

Moreover, our results are informative about the likely mode of transmission of *C. jejuni* to patients in our study. The genetic analysis identifies the source of infection, rather than the transmission route. The importance of livestock as a reservoir for human disease is consistent with food-borne transmission, but alternative pathways, such as ingestion of animal feces or contamination of water by human or animal waste, must also be considered. Our findings show that, while we can detect cases of human infection with isolates of an environmental or wild animal origin, such cases are rare, and this is surprising if pathways other than food-borne transmission are important. Therefore the dual observations that (i) livestock are a frequent source of human disease isolates and (ii) wild animals and the environment are not, strongly support the notion that preparation or consumption of infected meat and poultry is the dominant transmission route.

Transmission through the food chain can be controlled in a number of ways. Preventing cross-contamination of carcasses during processing is an effective measure [34] that can be achieved, for example, by minimizing meat contamination with animal feces, treating carcasses with antimicrobial agents, sterilizing equipment, and careful management of animals or flocks known to be infected. Meat products can be treated directly, for example by freezing or irradiation [35]. Promoting better standards of food hygiene during preparation and cooking is also an effective measure [32],[34].

Our results pertain to sporadic disease; we know that contamination of drinking water occasionally causes outbreaks [11]–[13]. The lack of evidence for pigs as a source of *C. jejuni* infection is consistent with their greater susceptibility to *C. coli* [36]. Since *C. coli* causes less than 10% of sporadic campylobacteriosis, pigs must be a less important source of infection than chicken and cattle.

We found considerable variation in the genetic make-up of *C. jejuni* populations sampled from similar sources (*e.g.* chicks *vs* chicken meat/offal) and between different populations from the same source type. This variation may reflect functional differences between *C. jejuni* even from closely related sources, or it may reflect stochastic differences in gene frequency over time or space. The epidemic process may increase variation in gene frequencies because hosts sampled locally are infected from the same source, causing non-independence within samples. We found our method was robust to this heterogeneity, but it is reasonable to think that inference would be improved by sensibly modeling the phenomenon. How to do so is unclear: one option is to split heterogeneous groups into further sub-categories, but that increases the number of parameters in the model and may reduce statistical efficiency or lead to over-fitting. Comprehensive sampling of putative source populations in parallel to human sampling is most desirable, and such studies are on-going by groups in Scotland, New Zealand and the USA.

Assigning the source of human isolates based on genotype has been attempted before in *C. jejuni*. Our results are in contrast to those of Champion *et al.* [18] who, using a Bayesian phylogenetic approach applied to comparative genomic hybridization data, found that *C. jejuni* isolates can be divided into livestock and non-livestock clades, with 55.7% of human isolates falling into the non-livestock clade. The existence of these clades was supported by high posterior probabilities, close to *Pr* = 1. The implications of such findings would be dramatic, however there are difficulties with the approach. The principal problem is that *C. jejuni* is known to be highly recombining which means that different genes, or even different parts of the same gene, will have different phylogenetic histories. Inferring a single phylogenetic tree for the whole genome is therefore a case of gross model mis-specification, and the resulting phylogeny is difficult to interpret in any meaningful way [37].

Many pathogens exist as weakly differentiated, genetically overlapping populations or strains between which there is frequent gene flow and within which there is frequent recombination. Such strains may be epidemiologically relevant, but it will be difficult to find stable, well-differentiated genetic markers, the standard tools of molecular epidemiology, to type them unambiguously. In this paper the method we developed addressed the problem in *C. jejuni* by assigning isolates to source populations probabilistically. We used a simple epidemiological model, in which we inferred the probability of infection with each source, to efficiently combine information over cases. That model could be readily extended in the general linear model framework to employ covariates, such as age, sex or host genotype, were they available.

In conclusion, we have used a novel population genetics approach to identify the source of infection of the zoonotic pathogen *Campylobacter jejuni*. We found that cases of human infection in our study were overwhelmingly attributable to bacteria characteristic of those colonizing animals farmed for meat and poultry, based on genetic similarity. We hope that demonstrating the importance of livestock as reservoirs of *Campylobacter* infectious to humans will add impetus to initiatives aimed at controlling food-borne pathogens.

## Materials and Methods

### Human Isolates

Stool samples were collected from 1,549 patients diagnosed with campylobacteriosis and notified through general practitioners and hospitals to the Preston Microbiology Services Laboratory in the Preston postcode district between January 1^st^ 2000 and December 31^st^ 2002. This covers an area of 968 km^2^, comprising 403,000 people at the 2001 census, consisting of both urban (Preston, Leyland, Chorley, Garstang) and rural (Ribble estuary and Ribble valley) districts. As is the norm with campylobacteriosis, the cases we studied were sporadic in nature; there was no evidence for outbreaks. We followed previously published methods for multilocus sequence typing *C. jejuni* [21],[38]. We obtained culturable, uncontaminated isolates of *Campylobacter* species from 1,353 samples, of which we identified 1,255 *C. jejuni*, 86 *C. coli* and 11 other species. One isolate tested positive for both *C. jejuni* and *C. coli* using the hippurate hydrolysis test and PCR. We fully sequenced all seven MLST loci (3,309 nucleotides in total) in 1,231 *C. jejuni* isolates, a sequencing success rate of 98%.

### Animal and Environmental Isolates

We collated 1,145 *C. jejuni* isolates of animal and environmental origin from ten previously published studies [7], [16], [21]–[28]. Where the sampling date was available, we excluded isolates sampled prior to 1990. We grouped the isolates by host or environmental origin as follows: chicken (515 isolates), cattle (282), sheep (160), pig (30), wild bird (44), wild rabbit (20), bathing beach (71) and environmental water sources (23). Table S1 gives a detailed breakdown of groups by source type and publication.

### Analysis of Molecular Variance

To analyze the genetic heterogeneity within each group, we estimated Φ-statistics using analyses of molecular variance (AMOVA [29]). Genetic distance between a pair of isolates was defined as the number of loci, out of seven, at which they differed. We defined sub-groups using detailed sampling information from each publication (Table S1). *E.g.* we defined isolates sampled from calves versus cows milk as separate sub-groups within the cattle group. Isolates sampled from the same source type in different studies were also defined as separate sub-groups. Significance was assessed by permutation test, using 999 permutations. To analyze genetic differentiation between groups, over and above within-group differentiation, we performed pairwise nested AMOVA. Significance was assessed in the same fashion.

### Source Attribution

The parameter of primary interest was the proportion, *F_j_*, of human cases attributable to source population *j* (*j* = 1…*n_g_*) where *n_g_* = 8 was the number of putative source populations, and 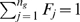. If we knew the source of each case, we could estimate *F* directly using the multinomial likelihood
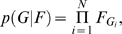
where *N* = 1,231 was the number of cases and *G_i_* was the source of origin for case *i*. Our approach was Bayesian, so the posterior probability distribution for *F*, upon which inference is based, would be
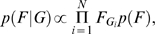
where *p*(*F*) is a prior probability distribution on the source attribution probabilities. We used a symmetric Dirichlet(1) prior on *F* in which all sources are considered equally likely *a priori*.

Of course we did not know *G*, so we used a genetic model of DNA sequence evolution to co-estimate the probable source of human isolates based on their genotypes, *H*, as follows,
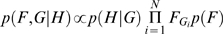
where *p*(*H*|*G*) is the likelihood of the source assignments *G* under our evolutionary model.

In our evolutionary model, we envisage the population of *C. jejuni* as a number of discrete islands: each source corresponds to a different island. Within each island the population is homogeneously mixing, and between islands there is migration. Migration rates may be higher between some islands than others, resulting in different levels of genetic differentiation. This is known as the migration matrix model [39], a generalization of Wright's island model. We modeled the generation of new alleles within each MLST locus using the infinite alleles model [40], and investigated two models of recombination between loci. In the first, the loci were assumed to be *unlinked* (inherited independently, or in linkage equilibrium), which is a computationally convenient but biologically unrealistic assumption. In the second, the loci were treated as *linked* (in linkage disequilibrium) using a model of recombination suitable for bacteria [41].

Human isolates were treated as a direct draw from one of the source populations. Therefore we assumed that the genotype of a human isolate would be representative of genotypes in the source population from which it was acquired. As a consequence, source attribution relies on the calculation of sampling probabilities; the likelihood that human isolate *i* was sampled from source population *j*. Unfortunately the complexity of the evolutionary model, in particular the *linked* model, renders direct calculation of the joint sampling probabilities *p*(*H*|*G*) impracticable, so we developed an approximation; full details of the approximation and the Markov Chain Monte Carlo sampler are provided in the Supplementary Methods (Text S1). To summarize, we used the animal and environmental isolates to estimate mutation, recombination and migration parameters. We then used these estimates together with all the genetic data (human and non-human genotypes) to jointly estimate the source attribution probabilities *F* and the source of human cases *G*. Except where stated otherwise, we used the mean of the posterior distribution for point estimates, and the (2.5%, 97.5%) quantiles of the posterior distribution for 95% credible intervals.

### Empirical Cross-Validation

We employed empirical cross-validation to assess various of aspects of our approach: (i) the adequacy of the approximations made in order to perform inference (ii) the robustness to violations of the modeling assumptions, such as genetic heterogeneity within groups, and (iii) the sensitivity to sample size differences between groups. During each iteration of the empirical cross-validation, we artificially removed the population of origin of half the 1,145 animal and environmental isolates at random. We then used the other half to infer their origin, and evaluated the performance of the two models. This procedure was repeated over 100 iterations.

We calculated several indicators of performance. The predicted proportion of isolates correctly assigned was calculated as
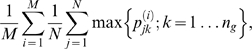
where there were *M* = 100 simulation, *N* = 572 pseudo-human cases, *n_g_* = 8 putative source populations, and 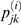 was the posterior probability that population *k* is the source of pseudo-human case *j* in simulation *i*. The bias in the estimate of the proportion of pseudo-human cases attributable to population *j* was calculated as
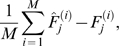
where 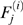 was the actual proportion attributable to population *j* in simulation *i*, and 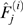 was the point estimate, *i.e.* the mean of the posterior distribution. The root mean squared error, which measures the variance of the point estimate, was calculated as
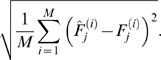



### Analysis of Robustness

As an additional test of robustness to the potentially confounding effects of genetic heterogeneity within the putative source populations, we repeated the source attribution analysis using subsets of the animal and environmental isolates, using the *linked* model only. The idea was to study the effect of removing whole sub-groups of isolates that were derived from the same source type or publication, as defined in Table S1. We conducted 100 simulations in order to generate samples of the non-human isolates in which 20% or more of the isolates were excluded, a whole sub-group at a time. The simulations were conducted as follows:

1. For source population *j*, sort the sub-groups (defined by Table S1) into descending order. Let *n* be the total number of isolates in population *j*, let *x*: = *n*/5 be the minimum number to exclude and let *i*: = 1 index the sub-groups.
2. With probability *x*/*n*, or if *n*−*n_i_*<*x*, remove all isolates of sub-group *i* and let *x*: = *x*−*n_i_*.
3. Let *i*: = *i*−1 and *n*: = *n*−*n_i_*. Repeat from (2) until *x*≤0.

On average, this procedure generated subsets in which 24.5% of isolates were excluded. Each of the 100 simulated subsets of the non-human isolates was used to infer the proportion of human cases attributable to each source. Figure S2 illustrates the difference in the simulation schemes between the empirical cross-validation and the analysis of robustness.

### Genotypes Unique to Humans

We performed two re-sampling procedures to compare the number of unique genotypes in human isolates to the number in other groups. The aim was to scrutinize two modeling assumptions: (i) that human isolates are merely a sample of *C. jejuni* isolates found in the putative source populations, and (ii) that the major source populations have been identified. In the first procedure, we removed one non-human group, *e.g.* chicken, from the “pool” of non-human isolates and calculated the number of unique genotypes by reference to the pool. We sampled a subset of human isolates, equal in size to the number of chicken isolates, and calculated the number of unique genotypes by reference to the same pool. We repeated the sampling of human isolates 100 times to generate a distribution for the number of genotypes unique to humans, which we compared to the number of genotypes unique to chicken. Because of assumption (i) we expect humans to exhibit fewer unique genotypes.

In the second re-sampling procedure, we removed half of the isolates belonging to a non-human group, *e.g.* chicken, leaving the rest in the pool in order to emulate the status of human isolates, which we assumed are merely a sample of isolates found in the non-human source populations. We sampled a subset of human isolates equal in number, and calculated the number of genotypes unique to chicken and humans, by reference to the same pool. We repeated the procedure 100 times to generate a distribution of the number of genotypes unique to chickens and humans. Violation of assumptions (i) or (ii) could lead to an excess of genotypes unique to humans.

### Data Deposition

All newly-sequenced multi-locus sequence types are available for download from pubMLST.org/campylobacter.

## Supporting Information

Figure S1 Migration and mutation probabilities in the animal and environmental samples. For each *C. jejuni* reservoir, the pie chart shows the predictive probability that a newly-sampled allele is a novel mutant (black segment) or identical to one already observed in the same or another population (colored segment: Chicken-yellow, Cattle-red, Sheep-blue, Pig-pink, Bird-green, Rabbit-purple, Sand-beige, Water-cyan). The estimated probability of recombination in each reservoir sample was 0.057, 0.048, 0.046, 0.15, 0.10, 0.061, 0.12 and 0.054 respectively.
(0.78 MB TIF)

Figure S2 The simulation schemes used for (A) empirical cross-validation and (B) analysis of robustness. Each row represents a non-human isolate; isolates are ordered vertically by source and sub-group (as defined by Table S1), and colored by group. In (A) blank spaces represent isolates assigned to the pseudo-human group. Their source was inferred from the remaining non-human isolates. In (B) blank spaces represent isolates that were excluded, whole sub-groups at a time, from inferring the source of human isolates.
(2.69 MB TIF)

Figure S3 Analysis of robustness. For each parameter (proportion of cases attributable to (A) chicken (B) cattle+sheep (C) cattle *vs.* sheep (D) bird (E) water (F) pig (G) rabbit (H) sand), the point estimate and the 95% credible interval is plotted for the analysis of 100 simulations and the full data. The results are ordered vertically by the point estimate, for which the posterior mean was used except in (C) where the posterior median was used. The red dot indicates the analysis of the full data.
(1.80 MB TIF)

Figure S4 The number of genotypes unique to humans. Two re-sampling procedures were performed (see Materials and Methods) to compare the number of genotypes unique to humans and other groups, controlling for sample size. The distribution of the number of unique genotypes is represented with box-and-whisker plots. (A) Humans exhibit fewer unique genotypes than non-human groups. (B) Humans exhibit no more unique genotypes than non-human groups that are partially represented in the pool of other non-human isolates.
(0.65 MB TIF)

Table S1 Source of animal and environmental isolates.
(0.11 MB DOC)

Table S2 Proportion of cases attributable to each source: summary of the posterior distribution of *F*.
(0.04 MB DOC)

Table S3 Posterior assignment probabilities by sequence type.
(0.57 MB DOC)

Text S1 Supplementary Methods.
(0.16 MB DOC)
