# Supplementary figures and images for: Tracing the Source of Campylobacteriosis
Source: PLoS Genet. 2008 Sep 26;4(9):e1000203. doi: 10.1371/journal.pgen.1000203 (PMC2538567; doi:10.1371/journal.pgen.1000203)

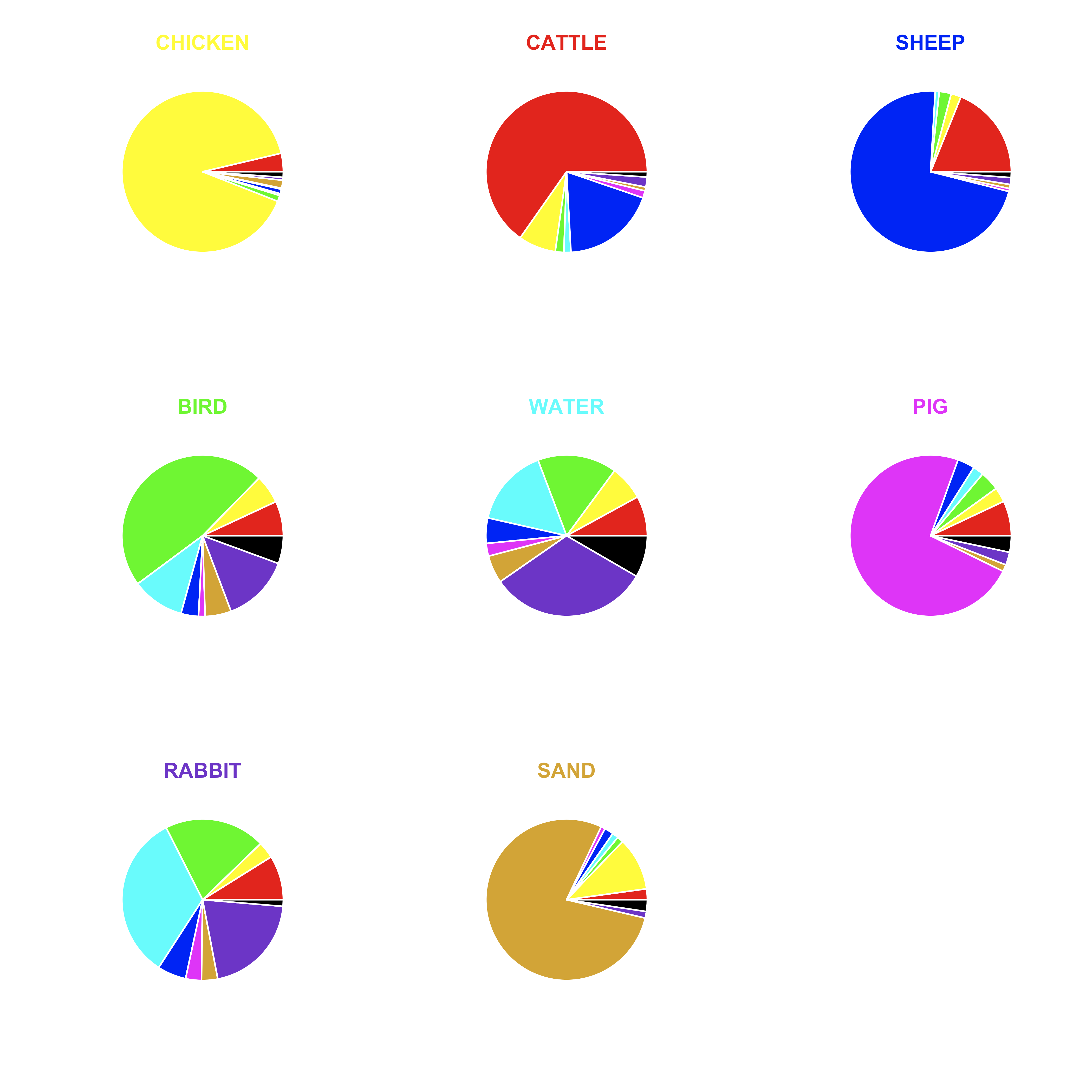

Supplement: Figure S1 — Migration and mutation probabilities in the animal and environmental samples. For each C. jejuni reservoir, the pie chart shows the predictive probability that a newly-sampled allele is a novel mutant (black segment) or identical to one already observed in the same or another population (colored segment: Chicken-yellow, Cattle-red, Sheep-blue, Pig-pink, Bird-green, Rabbit-purple, Sand-beige, Water-cyan). The estimated probability of recombination in each reservoir sample was 0.057, 0.048, 0.046, 0.15, 0.10, 0.061, 0.12 and 0.054 respectively. (0.78 MB TIF) [file pgen.1000203.s001.tif]

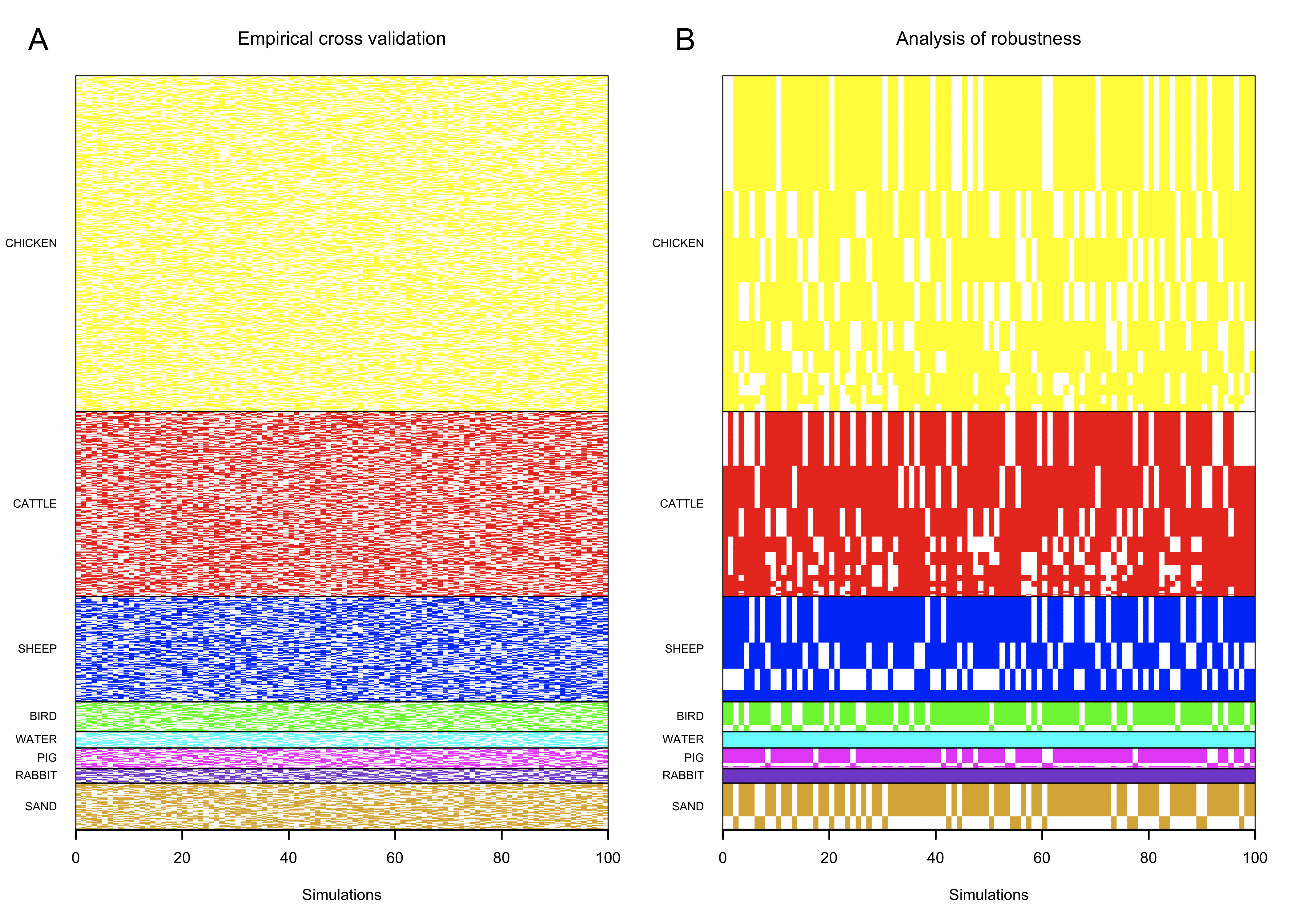

Supplement: Figure S2 — The simulation schemes used for (A) empirical cross-validation and (B) analysis of robustness. Each row represents a non-human isolate; isolates are ordered vertically by source and sub-group (as defined by Table S1), and colored by group. In (A) blank spaces represent isolates assigned to the pseudo-human group. Their source was inferred from the remaining non-human isolates. In (B) blank spaces represent isolates that were excluded, whole sub-groups at a time, from inferring the source of human isolates. (2.69 MB TIF) [file pgen.1000203.s002.tif]

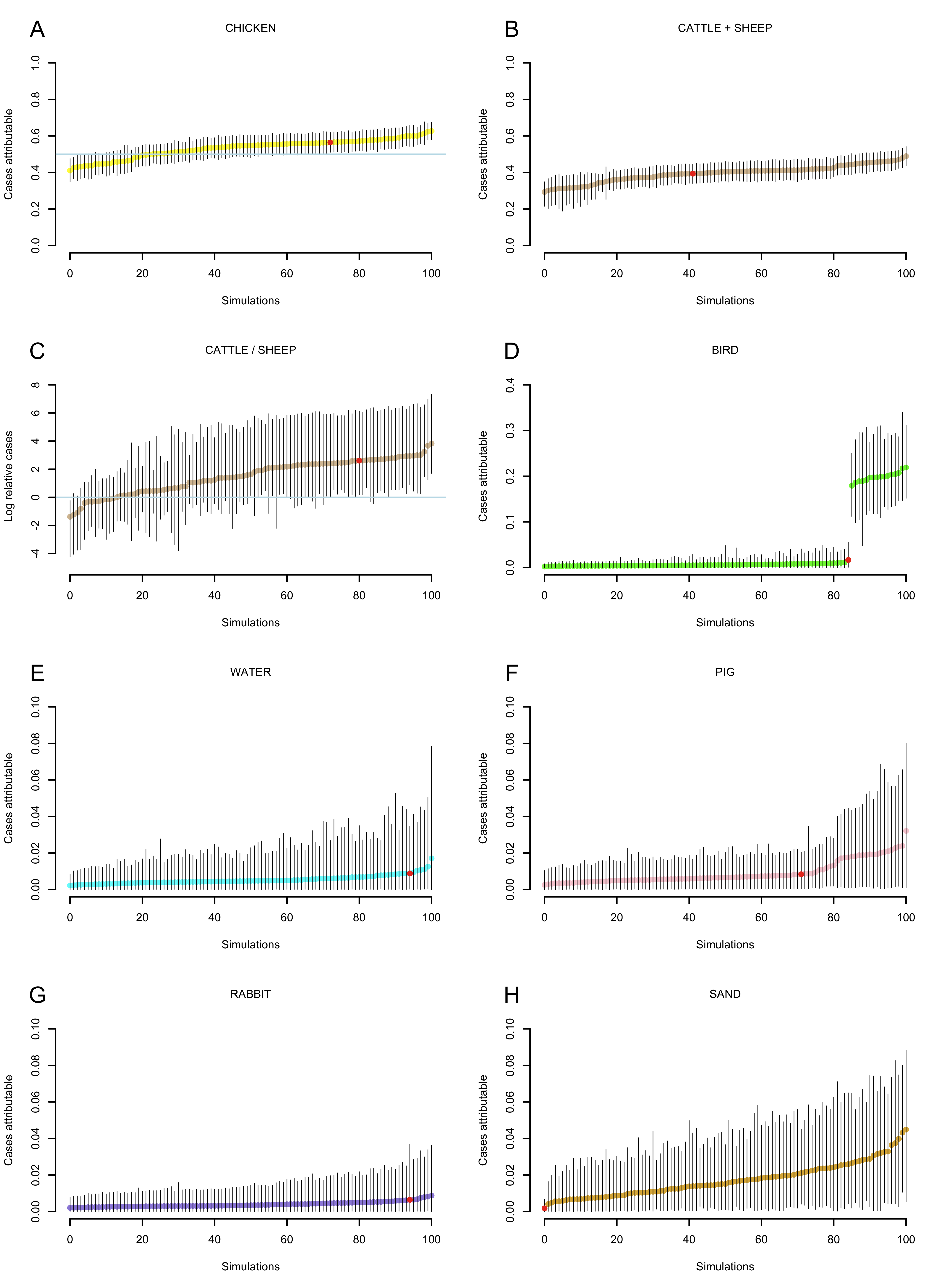

Supplement: Figure S3 — Analysis of robustness. For each parameter (proportion of cases attributable to (A) chicken (B) cattle+sheep (C) cattle vs. sheep (D) bird (E) water (F) pig (G) rabbit (H) sand), the point estimate and the 95% credible interval is plotted for the analysis of 100 simulations and the full data. The results are ordered vertically by the point estimate, for which the posterior mean was used except in (C) where the posterior median was used. The red dot indicates the analysis of the full data. (1.80 MB TIF) [file pgen.1000203.s003.tif]

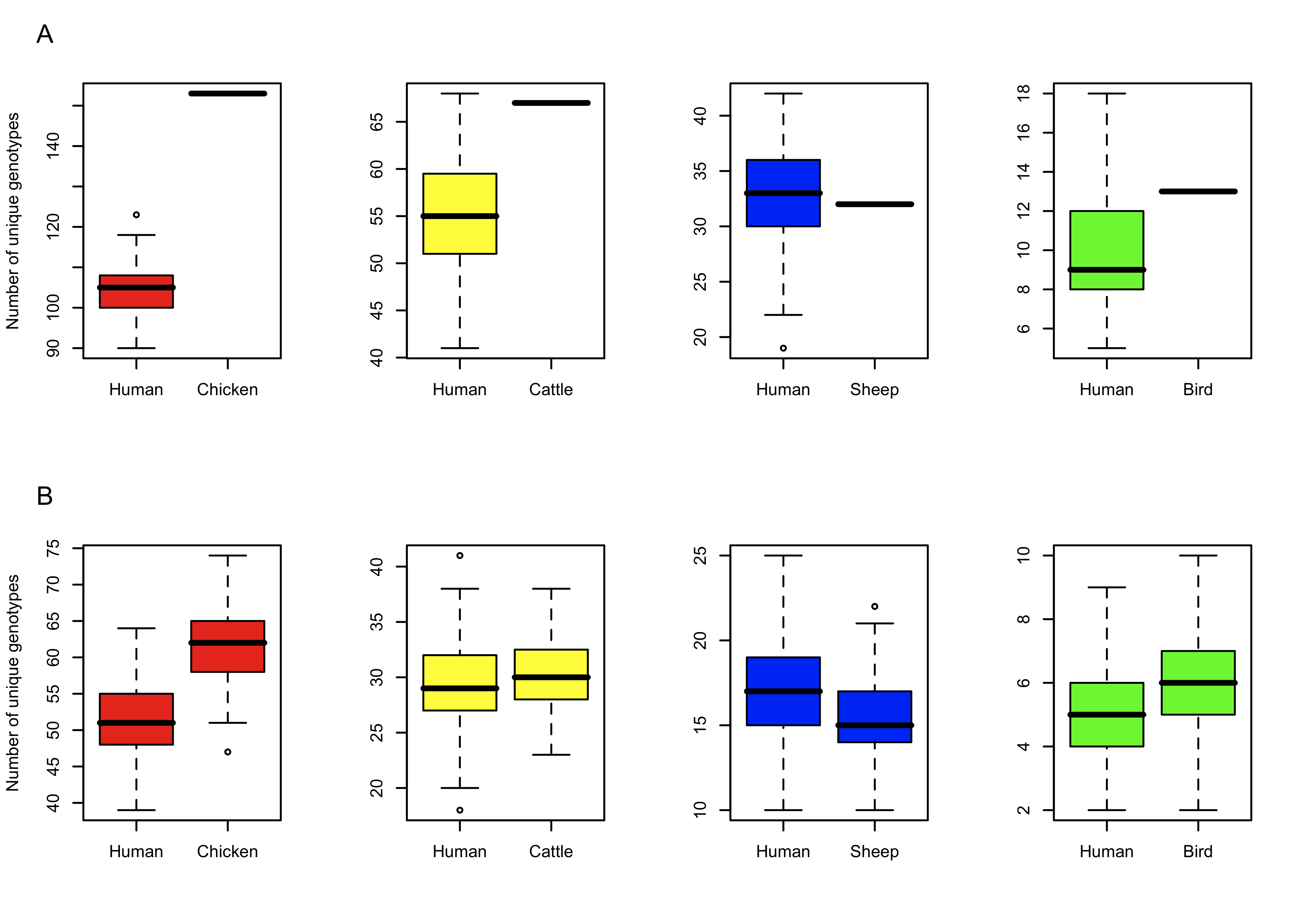

Supplement: Figure S4 — The number of genotypes unique to humans. Two re-sampling procedures were performed (see Materials and Methods) to compare the number of genotypes unique to humans and other groups, controlling for sample size. The distribution of the number of unique genotypes is represented with box-and-whisker plots. (A) Humans exhibit fewer unique genotypes than non-human groups. (B) Humans exhibit no more unique genotypes than non-human groups that are partially represented in the pool of other non-human isolates. (0.65 MB TIF) [file pgen.1000203.s004.tif]
