# Supplementary material for: Tracing the Source of Campylobacteriosis
Source: PLoS Genet. 2008 Sep 26;4(9):e1000203. doi: 10.1371/journal.pgen.1000203 (PMC2538567; doi:10.1371/journal.pgen.1000203)
Supplement: Table S1 — Source of animal and environmental isolates. (0.11 MB DOC) [file pgen.1000203.s005.doc]

## Table S1 Source of animal and environmental isolates

| LIVESTOCK: CHICKEN | | | | | |
| --- | --- | --- | --- | --- | --- |
| Reference | chick | chicken* | meat/offal | poultry | Total |
| Bull [21] |  | 177 |  |  | 177 |
| Colles [16] | 16 |  |  |  | 16 |
| Dingle [20] |  | 11 | 19 |  | 30 |
| Dingle [23] |  | 60 | 72 |  | 132 |
| Fitch [24] |  | 13 |  |  | 13 |
| Karenlampi[7] |  |  |  | 33** | 33 |
| Kinana [26] |  | 46 |  |  | 46 |
| Manning [27] | 68 |  |  |  | 68 |
| Total | 84 | 307 | 91 | 33 | 515 |
|  |  |  |  |  |  |
| LIVESTOCK: CATTLE | | | | | |
| Reference | calf | cattle* | cows milk | meat/offal | Total |
| Bull [21] |  | 2 |  |  | 2 |
| Clark [22] |  | 24 |  |  | 24 |
| Colles [16] | 9 | 15 |  |  | 24 |
| Dingle [20] | 1 | 7 | 3 |  | 11 |
| Dingle [23] | 1 | 9 |  | 44 | 54 |
| French [25] |  | 82 |  |  | 82 |
| Karenlampi[7] |  | 20 |  |  | 20 |
| Manning [27] |  | 65 |  |  | 65 |
| Total | 11 | 224 | 3 | 44 | 282 |
|  |  |  |  |  |  |
| LIVESTOCK: SHEEP | | | | |  |
| Reference | lamb | meat/offal | sheep* | Total |  |
| Colles [16] | 9 |  | 33 | 42 |  |
| Dingle [20] |  |  | 1 | 1 |  |
| Dingle [23] |  | 70 | 2 | 72 |  |
| French [25] |  |  | 5 | 5 |  |
| Manning [27] |  |  | 40 | 40 |  |
| Total | 9 | 70 | 81 | 160 |  |
|  |  |  |  |  |  |
| LIVESTOCK: PIG | | | |  |  |
| Reference | meat/offal | pig* | Total |  |  |
| Dingle [20] |  | 2 | 2 |  |  |
| Dingle [23] | 5 | 1 | 6 |  |  |
| Manning [27] |  | 22 | 22 |  |  |
| Total | 5 | 25 | 30 |  |  |
|  |  |  |  |  |  |
| WILD ANIMALS: BIRD | | | |  |  |
| Reference | bird* | starling | Total |  |  |
| Colles [16] |  | 9 | 9 |  |  |
| French [25] | 35 |  | 35 |  |  |
| Total | 35 | 9 | 44 |  |  |
|  |  |  |  |  |  |
| WILD ANIMALS: RABBIT | | |  |  |  |
| Reference | rabbit | Total |  |  |  |
| French [25] | 20 | 20 |  |  |  |
| Total | 20 | 20 |  |  |  |
|  |  |  |  |  |  |
| ENVIRONMENT: SAND | | | |  |  |
| Reference | sand [bathing beach] | | Total |  |  |
| Dingle [20] | 21 | | 21 |  |  |
| Dingle [23] | 50 | | 50 |  |  |
| Total | 71 | | 71 |  |  |
|  |  |  |  |  |  |
| ENVIRONMENT: WATER | | |  |  |  |
| Reference | water | Total |  |  |  |
| French [25] | 23 | 23 |  |  |  |
| Total | 23 | 23 |  |  |  |

* These generic sub-groups include isolates for which no further categorization was available

** 29 of the 33 isolates were chicken and 4 turkey, but the identities were not specified
