# Supplementary material for: Tracing the Source of Campylobacteriosis
Source: PLoS Genet. 2008 Sep 26;4(9):e1000203. doi: 10.1371/journal.pgen.1000203 (PMC2538567; doi:10.1371/journal.pgen.1000203)
Supplement: Table S2 — Proportion of cases attributable to each source: summary of the posterior distribution of F. (0.04 MB DOC) [file pgen.1000203.s006.doc]

## *Table S2 Proportion of cases attributable to each source: summary of the posterior distribution of* F

|  | Proportion of cases attributable to source (*F*) | | | | | | | |
| --- | --- | --- | --- | --- | --- | --- | --- | --- |
|  | Chicken | Cattle | Sheep | Pig | Bird | Rabbit | Sand | Water |
| Mean | 0.565 | 0.350 | 0.043 | 0.008 | 0.017 | 0.006 | 0.002 | 0.009 |
| Median | 0.565 | 0.361 | 0.027 | 0.008 | 0.012 | 0.003 | 0.001 | 0.006 |
| Standard Deviation | 0.027 | 0.056 | 0.047 | 0.005 | 0.015 | 0.010 | 0.002 | 0.009 |
| 2.5% quantile | 0.511 | 0.208 | 0.001 | 0.000 | 0.001 | 0.000 | 0.000 | 0.000 |
| 97.5% quantile | 0.618 | 0.432 | 0.175 | 0.020 | 0.055 | 0.037 | 0.007 | 0.035 |
