# Supplementary material for: Tracing the Source of Campylobacteriosis
Source: PLoS Genet. 2008 Sep 26;4(9):e1000203. doi: 10.1371/journal.pgen.1000203 (PMC2538567; doi:10.1371/journal.pgen.1000203)
Supplement: Table S3 — Posterior assignment probabilities by sequence type. (0.57 MB DOC) [file pgen.1000203.s007.doc]

## Table S3 Posterior assignment probabilities by sequence type

| Sequence | Number of | Posterior probability of source of infection | | | | | | | |
| --- | --- | --- | --- | --- | --- | --- | --- | --- | --- |
| Type | Cases | Chicken | Cattle | Sheep | Pig | Bird | Rabbit | Sand | Water |
| 5 | 6 | 0.149 | 0.485 | 0.032 | 0.284 | 0.011 | 0.021 | 0.001 | 0.017 |
| 8 | 1 | 0.260 | 0.685 | 0.045 | 0.002 | 0.005 | 0.002 | 0.000 | 0.002 |
| 11 | 2 | 0.540 | 0.437 | 0.018 | 0.001 | 0.002 | 0.001 | 0.000 | 0.001 |
| 19 | 34 | 0.398 | 0.473 | 0.121 | 0.001 | 0.002 | 0.002 | 0.000 | 0.002 |
| 21 | 149 | 0.255 | 0.660 | 0.069 | 0.001 | 0.007 | 0.005 | 0.000 | 0.004 |
| 22 | 18 | 0.331 | 0.534 | 0.128 | 0.001 | 0.002 | 0.002 | 0.000 | 0.002 |
| 25 | 1 | 0.946 | 0.049 | 0.002 | 0.000 | 0.001 | 0.000 | 0.000 | 0.001 |
| 38 | 2 | 0.078 | 0.838 | 0.060 | 0.001 | 0.006 | 0.011 | 0.000 | 0.005 |
| 42 | 10 | 0.075 | 0.718 | 0.168 | 0.002 | 0.014 | 0.013 | 0.000 | 0.010 |
| 43 | 1 | 0.525 | 0.450 | 0.020 | 0.001 | 0.002 | 0.001 | 0.000 | 0.001 |
| 44 | 1 | 0.943 | 0.052 | 0.002 | 0.000 | 0.001 | 0.000 | 0.000 | 0.001 |
| 45 | 59 | 0.743 | 0.213 | 0.015 | 0.001 | 0.009 | 0.011 | 0.000 | 0.008 |
| 47 | 6 | 0.775 | 0.138 | 0.015 | 0.002 | 0.020 | 0.007 | 0.032 | 0.012 |
| 48 | 86 | 0.081 | 0.816 | 0.093 | 0.001 | 0.003 | 0.003 | 0.001 | 0.002 |
| 49 | 7 | 0.590 | 0.359 | 0.019 | 0.001 | 0.019 | 0.008 | 0.000 | 0.005 |
| 50 | 52 | 0.894 | 0.090 | 0.012 | 0.000 | 0.001 | 0.001 | 0.000 | 0.001 |
| 51 | 20 | 0.944 | 0.051 | 0.002 | 0.000 | 0.001 | 0.000 | 0.000 | 0.001 |
| 52 | 8 | 0.592 | 0.289 | 0.114 | 0.001 | 0.002 | 0.002 | 0.000 | 0.002 |
| 53 | 58 | 0.288 | 0.599 | 0.061 | 0.002 | 0.024 | 0.013 | 0.000 | 0.014 |
| 61 | 24 | 0.087 | 0.789 | 0.108 | 0.001 | 0.004 | 0.005 | 0.000 | 0.004 |
| 63 | 1 | 0.853 | 0.131 | 0.015 | 0.000 | 0.001 | 0.000 | 0.000 | 0.000 |
| 66 | 2 | 0.143 | 0.741 | 0.110 | 0.002 | 0.002 | 0.001 | 0.001 | 0.001 |
| 86 | 1 | 0.933 | 0.060 | 0.004 | 0.000 | 0.001 | 0.000 | 0.000 | 0.001 |
| 97 | 1 | 0.780 | 0.158 | 0.014 | 0.001 | 0.029 | 0.011 | 0.000 | 0.007 |
| 104 | 64 | 0.926 | 0.068 | 0.003 | 0.000 | 0.001 | 0.000 | 0.000 | 0.001 |
| 122 | 1 | 0.943 | 0.053 | 0.002 | 0.000 | 0.001 | 0.000 | 0.000 | 0.001 |
| 131 | 1 | 0.973 | 0.025 | 0.001 | 0.000 | 0.000 | 0.000 | 0.000 | 0.000 |
| 132 | 2 | 0.076 | 0.875 | 0.040 | 0.001 | 0.003 | 0.002 | 0.000 | 0.002 |
| 137 | 14 | 0.692 | 0.178 | 0.036 | 0.002 | 0.049 | 0.022 | 0.000 | 0.020 |
| 188 | 1 | 0.495 | 0.440 | 0.032 | 0.002 | 0.018 | 0.007 | 0.000 | 0.005 |
| 205 | 12 | 0.075 | 0.877 | 0.040 | 0.001 | 0.002 | 0.002 | 0.000 | 0.002 |
| 206 | 13 | 0.061 | 0.760 | 0.169 | 0.001 | 0.003 | 0.003 | 0.000 | 0.002 |
| 222 | 1 | 0.884 | 0.109 | 0.005 | 0.000 | 0.001 | 0.000 | 0.000 | 0.000 |
| 227 | 4 | 0.919 | 0.075 | 0.003 | 0.000 | 0.001 | 0.000 | 0.000 | 0.001 |
| 230 | 4 | 0.079 | 0.873 | 0.040 | 0.001 | 0.003 | 0.002 | 0.000 | 0.002 |
| 233 | 4 | 0.708 | 0.211 | 0.018 | 0.001 | 0.037 | 0.014 | 0.001 | 0.010 |
| 257 | 149 | 0.706 | 0.227 | 0.028 | 0.001 | 0.024 | 0.009 | 0.000 | 0.006 |
| 262 | 15 | 0.203 | 0.668 | 0.121 | 0.001 | 0.002 | 0.002 | 0.000 | 0.002 |
| 266 | 1 | 0.059 | 0.761 | 0.170 | 0.001 | 0.003 | 0.003 | 0.000 | 0.002 |
| 267 | 12 | 0.945 | 0.050 | 0.002 | 0.000 | 0.001 | 0.001 | 0.000 | 0.001 |
| 270 | 3 | 0.075 | 0.768 | 0.070 | 0.072 | 0.004 | 0.006 | 0.000 | 0.004 |
| 273 | 2 | 0.058 | 0.752 | 0.180 | 0.001 | 0.003 | 0.003 | 0.000 | 0.002 |
| 290 | 1 | 0.943 | 0.053 | 0.002 | 0.000 | 0.001 | 0.000 | 0.000 | 0.001 |
| 305 | 2 | 0.944 | 0.051 | 0.002 | 0.000 | 0.001 | 0.000 | 0.000 | 0.001 |
| 311 | 1 | 0.943 | 0.052 | 0.002 | 0.000 | 0.001 | 0.000 | 0.000 | 0.001 |
| 324 | 5 | 0.944 | 0.051 | 0.002 | 0.000 | 0.001 | 0.000 | 0.000 | 0.001 |
| 325 | 1 | 0.944 | 0.051 | 0.002 | 0.000 | 0.001 | 0.000 | 0.000 | 0.001 |
| 334 | 5 | 0.654 | 0.161 | 0.025 | 0.003 | 0.100 | 0.034 | 0.000 | 0.022 |
| 350 | 2 | 0.948 | 0.020 | 0.001 | 0.008 | 0.011 | 0.000 | 0.001 | 0.012 |
| 353 | 7 | 0.945 | 0.051 | 0.002 | 0.000 | 0.001 | 0.000 | 0.000 | 0.001 |
| 354 | 14 | 0.919 | 0.068 | 0.011 | 0.000 | 0.001 | 0.001 | 0.000 | 0.001 |
| 356 | 8 | 0.998 | 0.001 | 0.000 | 0.000 | 0.000 | 0.000 | 0.000 | 0.000 |
| 361 | 1 | 0.675 | 0.268 | 0.028 | 0.001 | 0.019 | 0.005 | 0.000 | 0.004 |
| 372 | 1 | 0.945 | 0.049 | 0.002 | 0.000 | 0.001 | 0.000 | 0.000 | 0.001 |
| 393 | 3 | 0.998 | 0.001 | 0.000 | 0.000 | 0.000 | 0.000 | 0.000 | 0.000 |
| 400 | 4 | 0.993 | 0.006 | 0.000 | 0.000 | 0.000 | 0.000 | 0.000 | 0.000 |
| 403 | 6 | 0.097 | 0.458 | 0.030 | 0.367 | 0.010 | 0.021 | 0.001 | 0.016 |
| 436 | 1 | 0.944 | 0.051 | 0.002 | 0.000 | 0.001 | 0.000 | 0.000 | 0.001 |
| 441 | 2 | 0.861 | 0.097 | 0.008 | 0.016 | 0.011 | 0.000 | 0.001 | 0.006 |
| 443 | 3 | 0.943 | 0.052 | 0.002 | 0.000 | 0.001 | 0.000 | 0.000 | 0.001 |
| 447 | 1 | 0.891 | 0.093 | 0.004 | 0.000 | 0.006 | 0.002 | 0.000 | 0.002 |
| 448 | 1 | 0.270 | 0.374 | 0.046 | 0.006 | 0.151 | 0.073 | 0.001 | 0.079 |
| 449 | 2 | 0.826 | 0.036 | 0.001 | 0.003 | 0.088 | 0.001 | 0.001 | 0.046 |
| 450 | 4 | 0.998 | 0.001 | 0.000 | 0.000 | 0.001 | 0.000 | 0.000 | 0.000 |
| 464 | 1 | 0.876 | 0.031 | 0.000 | 0.082 | 0.005 | 0.001 | 0.002 | 0.002 |
| 466 | 3 | 0.989 | 0.010 | 0.000 | 0.000 | 0.000 | 0.000 | 0.000 | 0.000 |
| 474 | 1 | 0.784 | 0.194 | 0.018 | 0.000 | 0.002 | 0.001 | 0.000 | 0.001 |
| 475 | 6 | 0.522 | 0.454 | 0.019 | 0.001 | 0.002 | 0.001 | 0.000 | 0.001 |
| 507 | 1 | 0.265 | 0.485 | 0.047 | 0.004 | 0.100 | 0.011 | 0.001 | 0.088 |
| 508 | 7 | 0.311 | 0.305 | 0.054 | 0.007 | 0.202 | 0.072 | 0.001 | 0.049 |
| 520 | 3 | 0.048 | 0.676 | 0.265 | 0.001 | 0.003 | 0.004 | 0.000 | 0.003 |
| 523 | 2 | 0.874 | 0.118 | 0.003 | 0.000 | 0.002 | 0.000 | 0.001 | 0.001 |
| 524 | 2 | 0.997 | 0.002 | 0.000 | 0.000 | 0.000 | 0.000 | 0.000 | 0.000 |
| 531 | 3 | 0.570 | 0.108 | 0.002 | 0.003 | 0.255 | 0.001 | 0.001 | 0.059 |
| 538 | 1 | 0.764 | 0.148 | 0.006 | 0.001 | 0.056 | 0.008 | 0.005 | 0.012 |
| 561 | 1 | 0.147 | 0.788 | 0.039 | 0.002 | 0.014 | 0.005 | 0.000 | 0.005 |
| 564 | 1 | 0.945 | 0.051 | 0.002 | 0.000 | 0.001 | 0.000 | 0.000 | 0.001 |
| 572 | 14 | 0.976 | 0.023 | 0.000 | 0.000 | 0.000 | 0.000 | 0.000 | 0.000 |
| 573 | 4 | 0.944 | 0.051 | 0.002 | 0.000 | 0.001 | 0.000 | 0.000 | 0.001 |
| 574 | 19 | 0.944 | 0.051 | 0.002 | 0.000 | 0.001 | 0.000 | 0.000 | 0.001 |
| 581 | 1 | 0.988 | 0.003 | 0.000 | 0.003 | 0.004 | 0.000 | 0.000 | 0.001 |
| 583 | 5 | 0.194 | 0.619 | 0.040 | 0.003 | 0.075 | 0.045 | 0.000 | 0.024 |
| 584 | 19 | 0.984 | 0.011 | 0.001 | 0.000 | 0.004 | 0.000 | 0.000 | 0.000 |
| 595 | 1 | 0.104 | 0.620 | 0.036 | 0.207 | 0.010 | 0.011 | 0.003 | 0.009 |
| 606 | 3 | 0.993 | 0.006 | 0.000 | 0.000 | 0.000 | 0.000 | 0.000 | 0.000 |
| 607 | 3 | 0.993 | 0.006 | 0.000 | 0.000 | 0.000 | 0.000 | 0.000 | 0.000 |
| 618 | 1 | 0.122 | 0.748 | 0.049 | 0.002 | 0.023 | 0.023 | 0.000 | 0.032 |
| 634 | 1 | 0.055 | 0.673 | 0.259 | 0.001 | 0.005 | 0.004 | 0.000 | 0.003 |
| 658 | 6 | 0.799 | 0.120 | 0.007 | 0.004 | 0.026 | 0.003 | 0.036 | 0.006 |
| 661 | 6 | 0.964 | 0.022 | 0.000 | 0.000 | 0.009 | 0.000 | 0.001 | 0.003 |
| 677 | 2 | 0.185 | 0.547 | 0.038 | 0.006 | 0.077 | 0.078 | 0.001 | 0.069 |
| 693 | 1 | 0.636 | 0.034 | 0.001 | 0.005 | 0.190 | 0.000 | 0.008 | 0.126 |
| 696 | 1 | 0.076 | 0.877 | 0.040 | 0.001 | 0.002 | 0.002 | 0.000 | 0.002 |
| 759 | 1 | 0.302 | 0.562 | 0.130 | 0.001 | 0.003 | 0.001 | 0.000 | 0.001 |
| 760 | 1 | 0.318 | 0.317 | 0.046 | 0.009 | 0.130 | 0.006 | 0.001 | 0.174 |
| 768 | 1 | 0.332 | 0.044 | 0.004 | 0.561 | 0.018 | 0.001 | 0.001 | 0.039 |
| 775 | 1 | 0.967 | 0.029 | 0.003 | 0.000 | 0.000 | 0.000 | 0.000 | 0.000 |
| 791 | 1 | 0.944 | 0.051 | 0.002 | 0.000 | 0.001 | 0.000 | 0.000 | 0.001 |
| 824 | 4 | 0.944 | 0.051 | 0.002 | 0.000 | 0.001 | 0.000 | 0.000 | 0.001 |
| 875 | 1 | 0.998 | 0.001 | 0.000 | 0.000 | 0.001 | 0.000 | 0.000 | 0.000 |
| 877 | 1 | 0.922 | 0.008 | 0.000 | 0.001 | 0.055 | 0.000 | 0.000 | 0.014 |
| 878 | 1 | 0.996 | 0.004 | 0.000 | 0.000 | 0.000 | 0.000 | 0.000 | 0.000 |
| 883 | 1 | 0.075 | 0.878 | 0.040 | 0.001 | 0.002 | 0.002 | 0.000 | 0.002 |
| 904 | 1 | 1.000 | 0.000 | 0.000 | 0.000 | 0.000 | 0.000 | 0.000 | 0.000 |
| 916 | 1 | 0.238 | 0.502 | 0.049 | 0.005 | 0.103 | 0.012 | 0.000 | 0.091 |
| 969 | 1 | 0.975 | 0.020 | 0.004 | 0.000 | 0.000 | 0.000 | 0.000 | 0.000 |
| 974 | 1 | 0.993 | 0.005 | 0.000 | 0.000 | 0.001 | 0.000 | 0.000 | 0.000 |
| 982 | 2 | 0.403 | 0.530 | 0.056 | 0.001 | 0.005 | 0.002 | 0.000 | 0.003 |
| 985 | 1 | 0.032 | 0.125 | 0.007 | 0.589 | 0.069 | 0.009 | 0.003 | 0.166 |
| 986 | 1 | 0.389 | 0.221 | 0.013 | 0.010 | 0.177 | 0.003 | 0.016 | 0.172 |
| 990 | 2 | 0.604 | 0.204 | 0.024 | 0.003 | 0.108 | 0.009 | 0.001 | 0.046 |
| 1076 | 1 | 0.891 | 0.088 | 0.004 | 0.003 | 0.009 | 0.001 | 0.001 | 0.004 |
| 1257 | 2 | 0.913 | 0.002 | 0.000 | 0.003 | 0.028 | 0.000 | 0.033 | 0.021 |
| 1286 | 1 | 0.016 | 0.006 | 0.002 | 0.041 | 0.345 | 0.005 | 0.009 | 0.577 |
| 1316 | 1 | 0.016 | 0.006 | 0.002 | 0.041 | 0.345 | 0.005 | 0.009 | 0.577 |
| 1361 | 1 | 0.067 | 0.628 | 0.057 | 0.191 | 0.020 | 0.006 | 0.001 | 0.031 |
| 1374 | 1 | 0.932 | 0.018 | 0.001 | 0.031 | 0.016 | 0.000 | 0.000 | 0.002 |
| 1461 | 1 | 0.960 | 0.033 | 0.002 | 0.000 | 0.003 | 0.001 | 0.000 | 0.001 |
| 1476 | 1 | 0.908 | 0.078 | 0.012 | 0.000 | 0.001 | 0.000 | 0.000 | 0.001 |
| 1509 | 1 | 0.657 | 0.197 | 0.014 | 0.003 | 0.053 | 0.011 | 0.001 | 0.064 |
| 1510 | 1 | 0.969 | 0.026 | 0.001 | 0.002 | 0.001 | 0.000 | 0.000 | 0.000 |
| 1511 | 1 | 0.959 | 0.028 | 0.001 | 0.001 | 0.006 | 0.000 | 0.000 | 0.004 |
| 1512 | 1 | 0.927 | 0.067 | 0.003 | 0.000 | 0.002 | 0.000 | 0.000 | 0.001 |
| 1513 | 1 | 0.842 | 0.105 | 0.008 | 0.006 | 0.030 | 0.005 | 0.000 | 0.004 |
| 1514 | 1 | 0.941 | 0.023 | 0.001 | 0.000 | 0.007 | 0.001 | 0.024 | 0.003 |
| 1515 | 1 | 0.065 | 0.457 | 0.022 | 0.369 | 0.027 | 0.009 | 0.001 | 0.050 |
| 1516 | 1 | 0.275 | 0.168 | 0.010 | 0.003 | 0.383 | 0.099 | 0.002 | 0.060 |
| 1517 | 3 | 0.898 | 0.072 | 0.011 | 0.001 | 0.007 | 0.001 | 0.001 | 0.009 |
| 1518 | 2 | 0.207 | 0.720 | 0.043 | 0.001 | 0.014 | 0.007 | 0.003 | 0.005 |
| 1519 | 5 | 0.970 | 0.027 | 0.001 | 0.000 | 0.001 | 0.000 | 0.000 | 0.000 |
| 1520 | 1 | 0.155 | 0.410 | 0.026 | 0.008 | 0.235 | 0.033 | 0.001 | 0.132 |
| 1521 | 1 | 0.666 | 0.238 | 0.003 | 0.080 | 0.009 | 0.001 | 0.001 | 0.002 |
| 1522 | 1 | 0.934 | 0.050 | 0.001 | 0.001 | 0.006 | 0.000 | 0.002 | 0.005 |
| 1523 | 1 | 0.099 | 0.811 | 0.030 | 0.002 | 0.037 | 0.011 | 0.000 | 0.008 |
| 1524 | 1 | 0.064 | 0.721 | 0.172 | 0.004 | 0.016 | 0.003 | 0.000 | 0.019 |
| 1525 | 1 | 0.379 | 0.129 | 0.001 | 0.024 | 0.260 | 0.004 | 0.027 | 0.175 |
| 1526 | 1 | 0.565 | 0.045 | 0.001 | 0.012 | 0.136 | 0.001 | 0.004 | 0.235 |
| 1527 | 1 | 0.981 | 0.016 | 0.000 | 0.000 | 0.002 | 0.000 | 0.001 | 0.000 |
| 1528 | 1 | 0.993 | 0.005 | 0.000 | 0.001 | 0.001 | 0.000 | 0.000 | 0.000 |
| 1709 | 1 | 0.974 | 0.007 | 0.000 | 0.000 | 0.006 | 0.000 | 0.008 | 0.005 |
| 1710 | 2 | 0.626 | 0.132 | 0.033 | 0.070 | 0.106 | 0.001 | 0.000 | 0.032 |
| 1712 | 1 | 0.778 | 0.152 | 0.007 | 0.002 | 0.045 | 0.009 | 0.000 | 0.006 |
| 1716 | 1 | 0.995 | 0.002 | 0.000 | 0.001 | 0.002 | 0.000 | 0.000 | 0.000 |
| 1721 | 1 | 0.990 | 0.001 | 0.000 | 0.002 | 0.003 | 0.000 | 0.000 | 0.004 |
| 1746 | 1 | 0.967 | 0.030 | 0.001 | 0.000 | 0.000 | 0.000 | 0.000 | 0.000 |
| 1852 | 1 | 0.928 | 0.061 | 0.001 | 0.000 | 0.009 | 0.000 | 0.001 | 0.001 |
| 1853 | 1 | 0.353 | 0.543 | 0.084 | 0.001 | 0.016 | 0.001 | 0.000 | 0.002 |
| 1854 | 1 | 0.065 | 0.896 | 0.034 | 0.000 | 0.003 | 0.001 | 0.000 | 0.001 |
| 1855 | 1 | 0.098 | 0.712 | 0.076 | 0.053 | 0.037 | 0.014 | 0.000 | 0.010 |
| 1856 | 1 | 0.293 | 0.581 | 0.121 | 0.001 | 0.002 | 0.001 | 0.000 | 0.001 |
| 1857 | 1 | 0.043 | 0.695 | 0.252 | 0.001 | 0.004 | 0.003 | 0.000 | 0.002 |
| 1858 | 1 | 0.990 | 0.008 | 0.000 | 0.000 | 0.001 | 0.000 | 0.000 | 0.001 |
| 1859 | 1 | 0.004 | 0.938 | 0.013 | 0.039 | 0.003 | 0.002 | 0.000 | 0.001 |
| 1860 | 1 | 0.918 | 0.080 | 0.001 | 0.000 | 0.001 | 0.000 | 0.000 | 0.000 |
| 1861 | 1 | 0.475 | 0.454 | 0.009 | 0.052 | 0.004 | 0.002 | 0.001 | 0.004 |
| 1862 | 1 | 0.548 | 0.388 | 0.057 | 0.001 | 0.004 | 0.001 | 0.000 | 0.001 |
| 1863 | 1 | 0.871 | 0.056 | 0.003 | 0.001 | 0.027 | 0.002 | 0.035 | 0.004 |
| 1864 | 1 | 0.969 | 0.028 | 0.001 | 0.000 | 0.002 | 0.000 | 0.000 | 0.000 |
| 1865 | 1 | 0.431 | 0.506 | 0.053 | 0.001 | 0.006 | 0.002 | 0.000 | 0.001 |
| 1866 | 1 | 0.973 | 0.021 | 0.001 | 0.002 | 0.001 | 0.000 | 0.000 | 0.000 |
| 1867 | 1 | 0.973 | 0.012 | 0.001 | 0.001 | 0.010 | 0.001 | 0.001 | 0.001 |
| 1868 | 1 | 0.591 | 0.319 | 0.030 | 0.004 | 0.038 | 0.008 | 0.000 | 0.009 |
| 1869 | 1 | 0.255 | 0.554 | 0.041 | 0.067 | 0.034 | 0.008 | 0.025 | 0.016 |
| 1870 | 1 | 0.956 | 0.038 | 0.002 | 0.000 | 0.003 | 0.000 | 0.001 | 0.001 |
| 1871 | 1 | 0.910 | 0.078 | 0.005 | 0.001 | 0.004 | 0.000 | 0.000 | 0.000 |
| 1872 | 1 | 0.131 | 0.683 | 0.124 | 0.005 | 0.033 | 0.003 | 0.001 | 0.020 |
| 1873 | 1 | 0.944 | 0.053 | 0.000 | 0.000 | 0.001 | 0.000 | 0.001 | 0.000 |
| 1874 | 1 | 0.029 | 0.821 | 0.144 | 0.001 | 0.002 | 0.002 | 0.000 | 0.001 |
| 1875 | 1 | 0.340 | 0.552 | 0.103 | 0.001 | 0.002 | 0.001 | 0.000 | 0.001 |
| 1876 | 1 | 0.989 | 0.010 | 0.000 | 0.000 | 0.000 | 0.000 | 0.000 | 0.000 |
| 1877 | 1 | 0.155 | 0.746 | 0.094 | 0.001 | 0.002 | 0.001 | 0.001 | 0.001 |
| 1878 | 1 | 0.265 | 0.639 | 0.083 | 0.001 | 0.007 | 0.002 | 0.001 | 0.002 |
| 1879 | 1 | 0.260 | 0.659 | 0.039 | 0.003 | 0.018 | 0.007 | 0.007 | 0.006 |
| 1880 | 1 | 0.680 | 0.254 | 0.018 | 0.002 | 0.030 | 0.008 | 0.000 | 0.007 |
| 1881 | 1 | 0.952 | 0.030 | 0.001 | 0.000 | 0.011 | 0.002 | 0.001 | 0.002 |
| 1882 | 1 | 0.973 | 0.021 | 0.005 | 0.000 | 0.001 | 0.000 | 0.000 | 0.000 |
| 1883 | 1 | 0.049 | 0.724 | 0.223 | 0.001 | 0.001 | 0.001 | 0.000 | 0.001 |
| 1884 | 2 | 0.586 | 0.133 | 0.016 | 0.015 | 0.190 | 0.001 | 0.000 | 0.058 |
| 1885 | 1 | 0.967 | 0.020 | 0.000 | 0.001 | 0.008 | 0.000 | 0.002 | 0.002 |
| 1886 | 1 | 0.768 | 0.149 | 0.002 | 0.001 | 0.049 | 0.001 | 0.001 | 0.029 |
| 1887 | 1 | 0.799 | 0.125 | 0.002 | 0.002 | 0.039 | 0.001 | 0.000 | 0.031 |
| 1888 | 1 | 0.853 | 0.143 | 0.001 | 0.000 | 0.002 | 0.000 | 0.000 | 0.000 |
| 1889 | 1 | 0.953 | 0.041 | 0.001 | 0.000 | 0.004 | 0.000 | 0.000 | 0.001 |
| 1890 | 1 | 0.198 | 0.302 | 0.018 | 0.005 | 0.392 | 0.052 | 0.000 | 0.033 |
| 1891 | 1 | 0.814 | 0.164 | 0.009 | 0.000 | 0.008 | 0.002 | 0.001 | 0.003 |
| 1892 | 1 | 0.058 | 0.902 | 0.034 | 0.001 | 0.003 | 0.001 | 0.000 | 0.001 |
| 1893 | 1 | 0.920 | 0.045 | 0.004 | 0.003 | 0.022 | 0.004 | 0.000 | 0.003 |
| 1894 | 1 | 0.830 | 0.139 | 0.007 | 0.002 | 0.014 | 0.004 | 0.000 | 0.004 |
| 1895 | 1 | 0.792 | 0.105 | 0.009 | 0.001 | 0.077 | 0.009 | 0.000 | 0.008 |
| 1896 | 1 | 0.860 | 0.126 | 0.002 | 0.000 | 0.007 | 0.001 | 0.000 | 0.003 |
| 1897 | 1 | 0.914 | 0.080 | 0.002 | 0.000 | 0.002 | 0.000 | 0.001 | 0.000 |
| 1898 | 1 | 0.317 | 0.401 | 0.015 | 0.209 | 0.028 | 0.015 | 0.002 | 0.012 |
| 1899 | 1 | 0.996 | 0.004 | 0.000 | 0.000 | 0.000 | 0.000 | 0.000 | 0.000 |
| 1900 | 3 | 0.948 | 0.047 | 0.001 | 0.002 | 0.001 | 0.000 | 0.001 | 0.000 |
| 1901 | 1 | 0.877 | 0.119 | 0.002 | 0.001 | 0.001 | 0.000 | 0.000 | 0.000 |
| 1902 | 1 | 0.997 | 0.002 | 0.000 | 0.000 | 0.000 | 0.000 | 0.000 | 0.000 |
| 1903 | 1 | 0.996 | 0.004 | 0.000 | 0.000 | 0.000 | 0.000 | 0.000 | 0.000 |
| 1904 | 1 | 0.993 | 0.006 | 0.000 | 0.000 | 0.000 | 0.000 | 0.000 | 0.000 |
| 1905 | 1 | 0.978 | 0.018 | 0.000 | 0.000 | 0.002 | 0.000 | 0.000 | 0.001 |
| 1906 | 1 | 0.899 | 0.019 | 0.001 | 0.007 | 0.039 | 0.000 | 0.001 | 0.034 |
| 1907 | 1 | 0.558 | 0.377 | 0.004 | 0.050 | 0.004 | 0.002 | 0.001 | 0.004 |
| 1908 | 1 | 0.863 | 0.123 | 0.002 | 0.000 | 0.007 | 0.001 | 0.000 | 0.003 |
| 1909 | 3 | 0.901 | 0.093 | 0.003 | 0.000 | 0.001 | 0.000 | 0.001 | 0.001 |
| 1910 | 1 | 0.722 | 0.106 | 0.005 | 0.126 | 0.013 | 0.003 | 0.001 | 0.024 |
| 1911 | 2 | 0.987 | 0.009 | 0.000 | 0.000 | 0.002 | 0.000 | 0.000 | 0.001 |
| 1912 | 1 | 0.998 | 0.001 | 0.000 | 0.000 | 0.000 | 0.000 | 0.000 | 0.000 |
| 1913 | 1 | 0.932 | 0.063 | 0.002 | 0.000 | 0.001 | 0.000 | 0.000 | 0.001 |
| 1914 | 1 | 0.992 | 0.008 | 0.000 | 0.000 | 0.000 | 0.000 | 0.000 | 0.000 |
| 1915 | 1 | 0.998 | 0.002 | 0.000 | 0.000 | 0.000 | 0.000 | 0.000 | 0.000 |
| 1916 | 1 | 1.000 | 0.000 | 0.000 | 0.000 | 0.000 | 0.000 | 0.000 | 0.000 |
| 1917 | 1 | 0.688 | 0.252 | 0.049 | 0.002 | 0.007 | 0.001 | 0.001 | 0.001 |
| 1918 | 1 | 0.574 | 0.353 | 0.037 | 0.001 | 0.026 | 0.005 | 0.001 | 0.004 |
| 1919 | 1 | 0.738 | 0.179 | 0.021 | 0.055 | 0.005 | 0.001 | 0.001 | 0.001 |
| 1920 | 1 | 0.433 | 0.516 | 0.024 | 0.001 | 0.020 | 0.002 | 0.003 | 0.002 |
| 1921 | 1 | 0.955 | 0.038 | 0.001 | 0.000 | 0.005 | 0.000 | 0.000 | 0.001 |
| 1922 | 2 | 0.551 | 0.387 | 0.029 | 0.001 | 0.023 | 0.005 | 0.000 | 0.004 |
| 1923 | 1 | 0.810 | 0.149 | 0.025 | 0.001 | 0.011 | 0.002 | 0.000 | 0.002 |
| 1924 | 1 | 0.833 | 0.093 | 0.006 | 0.001 | 0.051 | 0.010 | 0.000 | 0.007 |
| 1925 | 1 | 0.982 | 0.017 | 0.000 | 0.000 | 0.000 | 0.000 | 0.000 | 0.000 |
| 1926 | 1 | 0.431 | 0.421 | 0.136 | 0.006 | 0.002 | 0.001 | 0.001 | 0.001 |
| 1927 | 1 | 0.033 | 0.051 | 0.001 | 0.649 | 0.102 | 0.008 | 0.004 | 0.150 |
| 1928 | 1 | 0.998 | 0.001 | 0.000 | 0.000 | 0.000 | 0.000 | 0.001 | 0.000 |
| 1929 | 1 | 0.995 | 0.001 | 0.000 | 0.000 | 0.001 | 0.000 | 0.002 | 0.001 |
| 1930 | 1 | 0.991 | 0.008 | 0.000 | 0.000 | 0.000 | 0.000 | 0.000 | 0.000 |
| 1931 | 1 | 0.997 | 0.002 | 0.000 | 0.000 | 0.000 | 0.000 | 0.000 | 0.000 |
| 1932 | 1 | 0.992 | 0.005 | 0.000 | 0.000 | 0.001 | 0.000 | 0.000 | 0.001 |
| 1933 | 1 | 0.049 | 0.806 | 0.037 | 0.097 | 0.005 | 0.002 | 0.001 | 0.002 |
| 1934 | 1 | 0.013 | 0.838 | 0.144 | 0.003 | 0.001 | 0.001 | 0.000 | 0.001 |
| 1935 | 1 | 0.027 | 0.013 | 0.001 | 0.012 | 0.430 | 0.003 | 0.027 | 0.487 |
| 1936 | 1 | 0.793 | 0.197 | 0.003 | 0.000 | 0.004 | 0.001 | 0.000 | 0.001 |
| 1972 | 1 | 0.830 | 0.032 | 0.000 | 0.002 | 0.090 | 0.001 | 0.001 | 0.045 |
| 2035 | 1 | 0.975 | 0.021 | 0.000 | 0.000 | 0.002 | 0.000 | 0.000 | 0.000 |
| 2238 | 1 | 0.932 | 0.013 | 0.000 | 0.003 | 0.029 | 0.000 | 0.001 | 0.022 |
| 2251 | 1 | 0.757 | 0.222 | 0.014 | 0.001 | 0.004 | 0.001 | 0.001 | 0.001 |
| 2265 | 1 | 0.945 | 0.001 | 0.000 | 0.000 | 0.002 | 0.000 | 0.052 | 0.000 |
| 2266 | 1 | 0.055 | 0.608 | 0.030 | 0.270 | 0.009 | 0.014 | 0.001 | 0.012 |
| 2267 | 1 | 0.964 | 0.031 | 0.001 | 0.000 | 0.002 | 0.000 | 0.000 | 0.002 |
| 2268 | 1 | 0.956 | 0.017 | 0.000 | 0.002 | 0.011 | 0.000 | 0.001 | 0.013 |
| 2288 | 1 | 0.880 | 0.090 | 0.013 | 0.001 | 0.009 | 0.000 | 0.001 | 0.006 |
| 2364 | 1 | 0.979 | 0.005 | 0.000 | 0.002 | 0.004 | 0.000 | 0.009 | 0.002 |
| 2383 | 1 | 0.089 | 0.733 | 0.107 | 0.004 | 0.025 | 0.005 | 0.000 | 0.036 |
| 2927 | 1 | 0.821 | 0.060 | 0.002 | 0.015 | 0.046 | 0.001 | 0.002 | 0.055 |
| 2962 | 1 | 0.813 | 0.065 | 0.002 | 0.004 | 0.042 | 0.001 | 0.002 | 0.071 |
| 2963 | 1 | 0.320 | 0.617 | 0.029 | 0.004 | 0.023 | 0.003 | 0.000 | 0.003 |
| 2964 | 1 | 0.146 | 0.785 | 0.049 | 0.001 | 0.013 | 0.003 | 0.001 | 0.002 |
| 2965 | 1 | 0.999 | 0.001 | 0.000 | 0.000 | 0.000 | 0.000 | 0.000 | 0.000 |
| 2966 | 1 | 0.994 | 0.004 | 0.000 | 0.001 | 0.000 | 0.000 | 0.000 | 0.000 |
| 2967 | 1 | 0.998 | 0.000 | 0.000 | 0.000 | 0.001 | 0.000 | 0.000 | 0.000 |
| 2968 | 1 | 0.998 | 0.001 | 0.000 | 0.000 | 0.000 | 0.000 | 0.000 | 0.000 |
| 2970 | 1 | 0.052 | 0.775 | 0.164 | 0.003 | 0.002 | 0.002 | 0.000 | 0.002 |
| 2971 | 1 | 0.924 | 0.056 | 0.002 | 0.001 | 0.007 | 0.001 | 0.001 | 0.009 |
| 2972 | 1 | 0.963 | 0.032 | 0.001 | 0.000 | 0.002 | 0.000 | 0.000 | 0.002 |
| 2973 | 1 | 0.007 | 0.001 | 0.000 | 0.001 | 0.836 | 0.027 | 0.002 | 0.125 |
| 3075 | 1 | 0.057 | 0.872 | 0.067 | 0.001 | 0.001 | 0.001 | 0.000 | 0.001 |
| 3076 | 1 | 0.845 | 0.127 | 0.003 | 0.001 | 0.011 | 0.001 | 0.001 | 0.011 |
| 3118 | 1 | 0.922 | 0.058 | 0.002 | 0.001 | 0.007 | 0.001 | 0.001 | 0.009 |
