# Supplementary material for: Tracing the Source of Campylobacteriosis
Source: PLoS Genet. 2008 Sep 26;4(9):e1000203. doi: 10.1371/journal.pgen.1000203 (PMC2538567; doi:10.1371/journal.pgen.1000203)
Supplement: Text S1 — Supplementary Methods. (0.16 MB DOC) [file pgen.1000203.s008.doc]

## Text S1 Supplementary Methods

**Model**

Human cases of *C. jejuni* are assumed to have been contracted from one of the *n_g_* = 8 source populations. Each human isolate is thought to represent an indirect sample from one of these populations, and the DNA sequence reflects which population was the source of infection.

Patterns of genetic diversity within and between the source populations are modeled using the migration matrix model [39] in which the populations are shaped by the processes of mutation, migration and recombination. The processes of mutation and intra-genic recombination are modeled using the infinite alleles model [40], and inter-genic recombination using a model suitable for transformation in bacteria [41].

| **Notation – Data and Parameters** | |
| --- | --- |
| *X, Y* | Source population and genotype of isolates sampled directly from animals or the environment. |
| *G, H* | Source population (unknown) and genotype of isolates sampled from human cases. |
| *μ_i_* | Probability that an allele sampled from population *i* is a novel mutant. |
| *M_ij_* | Probability that an allele sampled from population *i* has previously been observed in population *j* (*i, j* = 1...*n_g_*). |
| *R_i_* | Per-locus probability of recombination in a genotype sampled from source population *i*. |
| *F* | The proportion of human cases contracted from each source population. |

The parameter of interest is the proportion, *F_i_*, of human cases attributable to source population *i* (*i* = 1...*n_g_*). If the source-of-origin of a human case, *G_j_* (*j* = 1...*N*), were known, *F* could be estimated directly using the multinomial distribution. As *G* is unknown, we need to impute it on the basis of the genetic similarity of the human isolates to the animal and environmental isolates.

Calculation of the likelihood function is computationally prohibitive except for a small number of populations and sequences [42,43], even without the added complexity of incorporating recombination. This leads us to make a number of approximations, which we detail here. Central to our approach is the approximation of the sampling formula (*sensu* Ewens [44]). The sampling formula is the likelihood of observing genotype *y* in source population *x*, having already observed a set of genotypes *Y* sampled from source populations specified by the vector *X*. In this case the genotype *y* is an allelic profile consisting of seven allele numbers. We employ two approximations to the sampling formula, *φ*_1_ and *φ*_2_. In the first, the seven loci are assumed to be unlinked. In the second, we model linkage disequilibrium between the loci.

*Unlinked model*. In the unlinked model, the *L* = 7 loci are assumed independent. The likelihood of sampling a particular allele at locus *l*, which we denote *y*(*^l^*), depends on the evolutionary processes of *de novo* mutation and migration of existing alleles between populations. Loosely speaking, we will use *μ_x_* and *M_xm_* to denote mutation and migration probabilities. Formally, let *μ_x_* be the probability of sampling an allele from population *x* that is a novel mutant. Let *M_xm_* be the probability of sampling an allele from population *x* that has already been observed in population *m*. Let be the frequency with which allele *j* at locus *l* has already been observed in those genotypes *Y* sampled from population *m*. So the probability of sampling allele *j* at locus *l* from population *x*, in the absence of mutation, is . The sampling formula for the unlinked model is therefore

(1)

*Linked model*. To model linkage disequilibrium, we begin by assuming that in the absence of mutation, migration and recombination, a genotype *y* sampled from population *x* will be the same as one already observed in the sample *Y*. Migration allows *y* to be a copy of a genotype from a population other than *x*. We denote this template genotype *c* (*c* = 1...*N* where *N* is the total sample size of *Y*). We use *N_i_* to denote the number of genotypes *Y* sampled from population *i*.

The processes of mutation and recombination mean that genotype *y* may contain novel alleles, or comprise a novel combination of existing alleles. Let *μ_x_* be the probability, per locus, that a genotype sampled from population *x* contains a novel mutant allele. Let *R_x_* be the probability, per locus, that a genotype sampled from population *x* has undergone recombination. The effect of recombination is to un-link that locus and allow the allele to be independently sampled. The sampling formula for the linked model is therefore

(2)

*Pseudo-likelihood*. Normally, the sampling formula would be a function of rates of mutation, migration and recombination. The probabilities *μ*, *M* and *R* would depend on these rates, and the number of previously observed sequences in each population, *N_i_*. However, we do not know how the relationship between the probabilities *M* and *R* and the rates changes as a function of the sample sizes, *N_i_*. To circumvent the problem, we use a pseudo-likelihood approach [45], in which the *N_i_*, and hence *μ*, *M* and *R*, are forced to be roughly equal in the two stages of inference: (i) estimation of the evolutionary parameters, and (ii) prediction of the unknown source populations of human cases. This approach also forces the sequences to be exchangeable under the model, unlike in the PAC approach [46].

The pseudo-likelihood for sequences of known origin was calculated as

, (3)

where *Y_\i_* indicates the elements of *Y* excluding *Y_i_*. The likelihood for sequences of unknown origin, *H*, was calculated as

(4)

where *G* is the vector of unknown source populations. Equation (4) assumes that the *H_i_*’s are independent of one another conditional on the other data and parameters.

**Inference**

Our approach was Bayesian. To obtain stable and sensible estimates of the parameters, we found that it was necessary to perform inference in two parts. We first estimated the evolutionary parameters *μ*, *M* and *R* from the sequences of known origin. We then used the posterior distribution of *μ*, *M* and *R* to infer the source of origin of the human sequences, and in so doing, estimate the proportion of human cases attributable to each source population.

In step (i) we obtained samples from the posterior distribution of *μ*, *M* and *R* using Markov Chain Monte Carlo (MCMC)

(5)

For the evolutionary parameters, we used the following priors. For each source population *i*, a Beta(1, *n_g_*) distribution was used for *μ_i_*, a symmetric Dirichlet(1) distribution was used for *M_i•_*, and a Beta(1, 1) distribution was used for *R_i_*. The vector of known source populations *X* was treated as an auxiliary variable, i.e. it was conditioned upon.

In step (ii) the evolutionary parameters were repeatedly drawn from their posterior distribution, and for each draw, samples from the conditional posterior distribution of *F* were obtained by an MCMC side-chain. Uncertainty in the source of human sequences, *G*, was accounted for by summing over the possible populations-of-origin. So for draw *j*,

(6)

This two-step procedure causes inference of *μ*, *M* and *R* to be based on the sequences of known origin alone. To combine information about *F* over the 1,231 human isolates we used a simple epidemiological model to specify the likelihood *p*(*G*|*F*) of source assignments prior to observing the genotypes, in which *F* was the parameter of a multinomial distribution. We used a symmetric Dirichlet(1) distribution for the prior distribution *p*(*F*), so that all sources were equally likely *a priori*.

The MCMC scheme was run twice for 100,000 iterations each, with a burn-in of 10,000 iterations. Following burn-in, 100 side-chains were initiated at regular intervals. Each side-chain was run for 11,000 iterations with a burn-in of 1,000 iterations. The two chains were compared to assess convergence, and merged to obtain final results.

**Markov Chain Monte Carlo**

All the parameters sampled using MCMC (*μ*, *M*, *R* and *F*) were constrained (e.g. $0<F_{i}<1$, $\sum F_{i}=1$) which can cause numerical problems leading to failure of the algorithm. To overcome this issue, equivalent reparameterizations were used in the MCMC that make use of the following relationship between the Beta/Dirichlet distribution and the Gamma distribution:

If *X_j_*, *j* = 1...*n* are independent Gamma random variables with shape parameters *α_j_* and scale parameter 1, and , *j* = 1...*n*, then the vector *Y* has the Dirichlet distribution with parameter vector *α*.

| Original prior | Reparameterization | Transformed prior |
| --- | --- | --- |
| *μ_i_* ~ Beta(1,*n_g_*) |  | $\mu_{i1}^{'}$ ~ Gamma(1,1), $\mu_{i2}^{'}$~ Gamma(*n_g_*,1) |
| *M_i_*· ~ Dirichlet(1) |  | $M_{\text{ij}}^{'}$ ~ Gamma(1,1), *j* = 1...*n_g_* |
| *R_i_* ~ Beta(1,1) |  | $R_{i1}^{'}$ ~ Gamma(1,1), $R_{i2}^{'}$ ~ Gamma(1,1) |
| *F* ~ Dirichlet(1) |  | $F_{j}^{'}$ ~ Gamma(1,1), *j* = 1...*n_g_* |

Only two types of Metropolis-Hastings move were implemented: a log-normal proposal in which one of the transformed parameters was perturbed by a random amount, and a switching proposal in which the values were switched between a pair of parameters belonging to the same original Beta (e.g. $\mu_{i1}^{'}$ and $\mu_{i2}^{'}$) or Dirichlet distribution (e.g. $M_{\text{ij}}^{'}$ and $M_{\text{ik}}^{'}$).

*Log-normal move*. For example, an update to $F_{j}^{''}$ from $F_{j}^{'}$ is proposed: Let . This move is accepted with probability

, (7)

where *p*(•) is the prior and *l*(•) is the likelihood. Here *σ_F_* is a tuning parameter that can be altered to improve mixing of the MCMC.

*Switching move*. For example, it is proposed to switch $R_{i1}^{'}$ with $R_{i2}^{'}$. Let $R_{i1}^{''}=R_{i2}^{'}$ and $R_{i2}^{''}=R_{i1}^{'}$. The symmetry of the proposal means that this move is accepted with probability

. (8)

The relative rate at which these moves are proposed, which can differ for the different parameters, are also tuning parameters that can be altered to improve mixing of the MCMC.

## Additional References

1. Bahlo M, Griffiths RC (2000) Inference from gene trees in a subdivided populations. Theor Popul Biol 57: 79-95.
2. Beerli P, Felsenstein J (2001) Maximum likelihood estimation of a migration matrix and effective population sizes in n subpopulations by using a coalescent approach. P Natl Acad Sci U S A 98: 4563-4568.
3. Ewens WJ (1972) The sampling theory of selectively neutral alleles. Theor Popul Biol 3: 87-112.
4. Besag J (1975) Statistical analysis of non-lattice data. The Statistician 24: 179-195.
5. Li N, Stephens M (2003) Modeling linkage disequilibrium and identifying recombination hotspots using single-nucleotide polymorphism data. Genetics 165: 2213-2233.
